# Glucose-lowering effect of berberine on type 2 diabetes: A systematic review and meta-analysis

**DOI:** 10.3389/fphar.2022.1015045

**Published:** 2022-11-16

**Authors:** Wenting Xie, Fugui Su, Guizhong Wang, Zichong Peng, Yaomin Xu, Yi Zhang, Ningning Xu, Kaijian Hou, Zhuping Hu, Yan Chen, Rongping Chen

**Affiliations:** ^1^ Department of Endocrinology, Zhujiang Hospital, Southern Medical University/The Second School of Clinical Medicine, Southern Medical University, Guangzhou, Guangdong, China; ^2^ Department of Endocrinology, Suixi Country People’s Hospital, Guangdong Medical University, Guangzhou, Guangdong, China; ^3^ Department of Critical Care Medicine, Zhujiang Hospital, Southern Medical University/The Second School of Clinical Medicine, Southern Medical University, Guangzhou, Guangdong, China; ^4^ Department of Endocrine and Metabolic Diseases, Longhu Hospital, The First Affifiliated Hospital of Medical College of Shantou University, Shantou, Guangdong, China; ^5^ Department of Endocrinology, Wengyuan Country People’s Hospital, Shaoguan, China; ^6^ Department of Reproductive Medicine, Center of Maternal and Child Health Hospital of Shaoguan City, Shaoguan, Guangdong, China

**Keywords:** berberine, type 2 diabetes, meta-analysis, safety, glucose-lowering effect

## Abstract

**Background:** Insulin secretory agents are commonly used to treat type 2 diabetes. However, traditional insulin secretory agents such as sulfonylureas and glinides have side effects of hypoglycemia. In recent years, researchers have discovered that berberine can inhibit the voltage-gated k^+^ channels of pancreatic β cell membrane and promote insulin secretion without causing hypoglycemia, because the glucose-lowering effects of berberine are only under hyperglycemic conditions or in a high-glucose-dependent manner. In order to shed light on the glucose-lowing effects of berberine in type 2 diabetes with different baseline fasting plasma glucose (FPG) and glycosylated hemoglobin (HbA1c), we conducted a meta-analysis of randomized controlled trials.

**Methods:** We searched eight databases, which included PubMed, EMBASE, Web of Science, the Cochrane Library, and the Chinese databases such as Sino-Med, China National Knowledge Infrastructure (CNKI), Wanfang Database, and VIP Database for Chinese Technical Periodicals, for randomized controlled trials, with berberine as the intervention and patients with type 2 diabetes mellitus as subjects, published up until November 2021. We analyzed the glucose-lowing effects of berberine, including its effects on FPG, HbA1c and 2-h plasma blood glucose (2hPBG), by calculating weighted mean differences (WMD) and 95% confidence interval (CI). To assess the safety of berberine, we analyzed the incidence of total adverse events and hypoglycemia by calculating relative risk (RR) and 95% CI.

**Results:** Thirty-seven studies involving 3,048 patients were included in the meta-analysis. The results showed that berberine could reduce FPG (WMD = -0.82 mmol/L, 95% CI (-0.95, -0.70)), HbA1c (WMD = -0.63%, 95% CI (-0.72, -0.53)), and 2hPBG (WMD = -1.16 mmol/L, 95% CI (-1.36, -0.96)), with all results being statistically significant. Subgroup analyses revealed that the glucose-lowering effect of berberine was associated with baseline mean FPG and HbA1c in type 2 diabetes. In addition, berberine alone or in combination with oral hypoglycemic agents (OHAs) in the treatment of T2DM did not significantly increase the incidence of total adverse events (RR = 0.73, 95% CI (0.55, 0.97), *p* = 0.03) and the risk of hypoglycemia (RR = 0.48, 95% CI (0.21, 1.08), *p* = 0.08).

**Conclusion:** Berberine has a glucose-lowering effect, which is related to the baseline FPG and HbA1c levels of patients. Treatment with berberine may be safe since it does not increase the incidence of total adverse events and the risk of hypoglycemia.

**Systematic Review Registration:** https://www.crd.york.ac.uk/prospero/display_record.php?RecordID=292975, identifier CRD42021292975.

## 1 Introduction

According to the International Diabetes Federation (IDF) Diabetes Atlas, 10th edition, the global prevalence of diabetes among people between the ages of 20 and 79 years in 2021 was 10.5%, which is predicted to rise to 12.2% in 2045. Health expenditures associated with diabetes in the world in 2021 were about $966 billion, which are expected to increase to $1,054 billion by 2045 (Sun H. et al., 2022). There is growing evidence that chronic inflammation and some metabolic diseases are associated with insulin resistance, type 2 diabetes mellitus (T2DM) and systemic diabetic complications (Bray et al., 2021; Leite et al., 2021).

adays, oral hypoglycemic agents (OHAs) are commonly used in the treatment of T2DM, which can decrease blood glucose levels effectively. They mainly include the following categories: Sulfonylureas, glinides, biguanides, thiazolidinediones, α-glucosidase inhibitors, dipeptidyl peptidase-4 (DPP-4) inhibitors, glucagon-like peptide 1 (GLP-1) agonists and sodium-glucose transporter-2 (SGLT2) inhibitors. Increasing insulin secretion is regarded as one of the important strategies of treating T2DM. Drugs such as sulfonylureas and glinides inhibit the adenosine triphosphate (ATP) -sensitive potassium channel (K_ATP_) in pancreatic β cells, leading to the slow depolarization of pancreatic islet β-cell membrane and the inward flow of calcium ions, which in turn stimulates the release of insulin (Nagy et al., 2018). However, it is worth noting that these two classes of drugs may lead to severe hypoglycemia, which can cause 4 to 10 percent of disease-related deaths (Chang et al., 2020). Insulin and insulin secretagogues have been reported to increase the risk of developing breast, liver, pancreatic, and colorectal cancer (Tokajuk et al., 2015). Considering the impact of the adverse reactions potentially resulting from OHAs, many studies have investigated the relationship between T2DM and berberine (BBR).

In China, a classic Chinese herbal formula that includes BBR is a good remedy for diabetes. BBR is an isoquinoline alkaloid that is extracted from traditional Chinese medicines such as Cortex phellodendri (Huangbai) and Rhizoma coptidis (Huanglian) (Wu et al., 2020). Studies have shown that BBR has hyperglycemia-dependent glucose-lowering effects. Because BBR, as an insulinotropic agent, directly binds to KCNH6 potassium channels and reduces KCNH6 currents by accelerating channel closure, which prolongs the high glucose-dependent cell membrane depolarization and ultimately promotes insulin secretion (Zhao et al., 2021). The pharmacological mechanism of BBR implies that it acts as a glucose-lowering agent at high blood glucose levels, but this effect is not apparent in people with normal blood glucose, which greatly reduces the probability of hypoglycemia and provides a significant therapeutic advantage over OHAs. Recent meta-analyses (Dong et al., 2012; Lan et al., 2015; Liang et al., 2019; Guo et al., 2021) have all shown that BBR is effective in the treatment of T2DM. However, the latest two meta-analyses (Liang et al., 2019; Guo et al., 2021) showed obvious heterogeneity after including more literature than the earliest two meta-analyses (Dong et al., 2012; Lan et al., 2015), especially when including the literature on fasting plasma glucose (FPG). After a subgroup analysis, the problem of obvious heterogeneity still existed, which questioned the effectiveness of BBR. We believe that the obvious heterogeneity in the previous meta-analyses may stem from differences in the baseline mean FPG and glycosylated hemoglobin (HbA1c) of the patients they included.

Consequently, to illustrate the glucose-lowering effects of BBR in T2DM patients with different baseline mean FPG and HbA1c, which could provide references and recommendations for the clinical use of BBR, we systemically searched and analyzed all randomized controlled trials (RCTs) to perform a systematic literature review and a meta-analysis.

## 2 Methods

The review protocol was registered on PROSPERO (No: CRD42021292975).

### 2.1 Search strategy and study selection

Two investigators independently searched the following databases from study inception to November 2021: PubMed, EMBASE, Web of Science, the Cochrane Library, and the Chinese databases: Sino-Med, China National Knowledge Infrastructure (CNKI), Wanfang Database and VIP Database for Chinese Technical Periodicals. The search terms were used as follows [“Berberine” OR “Huangliansu” OR “Xiaobojian”] AND [“diabetes” OR “type 2 diabetes” OR “type 2 diabetes mellitus” OR “type II diabetes” OR “type II diabetes mellitus” OR “T2DM” OR “DM” OR “non-insulin-dependent diabetes mellitus”]. There was no restrictions on publication language or publication date, and the reference lists of included literature and related meta-analyses were manually searched to meet higher recall and precision ratio. PICOS of included studies was shown in Table 1. All articles were selected preliminarily by the title and abstract. For those that could not be judged by title or abstract, we read the full text.

**TABLE 1:** The PICOS of our study.

**PICOS terms**		**Details**
Population		T2DM patients of all ages and genders were included in our study. The criteria is base on *Chinese Guidelines for the prevention and treatment of Type 2 Diabetes Mellitus* or *1999 World Health Organization criteria.*
Interventions & Comparators		The Interventions group included the patients who were treated with berberine alone or in combination with other OHAs. The Comparators group included the patients who were treated with blank, placebo, lifestyle intervention or OHAs. In conclusion, only two situations fitted our purpose of exploring the effect of berberine, which are berberine vs. blank/placebo/lifestyle intervention and berberine combined with other OHAs vs. OHAs. If the study design didn’t include these two combinations, the study should be excluded. For example,
		1) Study A: berberine vs. placebo, the ‘berberine’ group is considered as intervention group, the ‘placebo’ group is considered as comparator group;
		2) Study B: berberine + metformin vs. berberine vs. metformin, the ‘berberine + metformin’ group is considered as intervention group, the ‘metformin’ group is considered as comparator group;
		3) Study C: berberine vs. sulfonylureas, excluded;
		4) Study D: berberine + metformin vs. berberine, excluded.
Outcomes	Primary	Fasting Plasma Glucose (FPG).
	Secondary	2-hour postprandial blood glucose (2hPBG), Glycated Hemoglobin (HbA1c), the incidence of total adverse events and hypoglycemia.
Study design		The studies were included if they met the following criteria:
		1) Met the criteria above in this table;
		2) Reported the baseline of FPG, 2hPBG or HbA1c and also reported them as an outcome indicator;
		3) Randomized controlled trials.
		The exclusion criteria were:
		1) the exact means and standard deviations of FPG, 2hPBG, HbA1c before and after treatment were not reported completely in article;
		2) T2DM with other chronic diseases or diabetic complications;
		3) The animal experiments;
		4) Non-RCT experiments, conference articles, reviews and duplicate publications

### 2.2 Data extraction and quality assessment

Two researchers independently extracted and cross-checked the information from selected articles, which included the published information, study design, patient information and the outcomes including FPG, 2hPBG, HbA1c, the incidence of total adverse events and hypoglycemia. The Cocrane Risk of Bias Tool was used to evaluate the quality of the included literature. The items included random sequence generation (selection bias), allocation concealment (selection bias), blinding of participants and personnel (performance bias), blinding of outcome assessment (detection bias), incomplete outcome data (attrition bias), selective reporting (reporting bias) and other bias. Each item was categorized as low/unclear/high risk of bias.

### 2.3 Data synthesis and analysis

The continuous variables were presented by calculating weighted mean differences (WMD) and 95% confidence interval (CI), whereas the risk ratio (RR) and its 95%CI were used for dichotomous outcomes. To assess the statistical heterogeneity among the included studies, we used I^2^ statistics and Cochrane’s Q test. When *p* > 0.1 in Cochrane’s Q test or I^2^ statistics <50%, a fixed effect model was chosen. When *p* < 0.1 or I^2^ statistics >50%, it showed that heterogeneity was significant, and we performed subgroup analyses according to the basis of patients’ FPG and HbA1c levels before treatment to investigate the source of heterogeneity. If the heterogeneity still existed, we used random effect model to explain the results cautiously. In order to test the stability of the research, sensitivity analyses were conducted to assess changes in FPG, the primary outcome, by excluding reports of apparently poor quality and eliminating studies with too short treatment duration (≤3w). Publication bias was evaluated using a funnel plot and Egger’s Test. All statistical analyses were performed using Revman version 5.3 and Stata version 12.0.

### 2.4 The quality of evidence

We rated the quality of the evidences of our meta-analysis with the grading of recommendations, assessment, development and evaluation (GRADE) approach, using the online tool named GRADEpro GDT ( *https://gdt.gradepro.org* ). Ratings were based on the GRADE handbook ( *https://gdt.gradepro.org/app/handbook/handbook.html* ).

## 3 Results

### 3.1 Literature search

**FIGURE 1:**
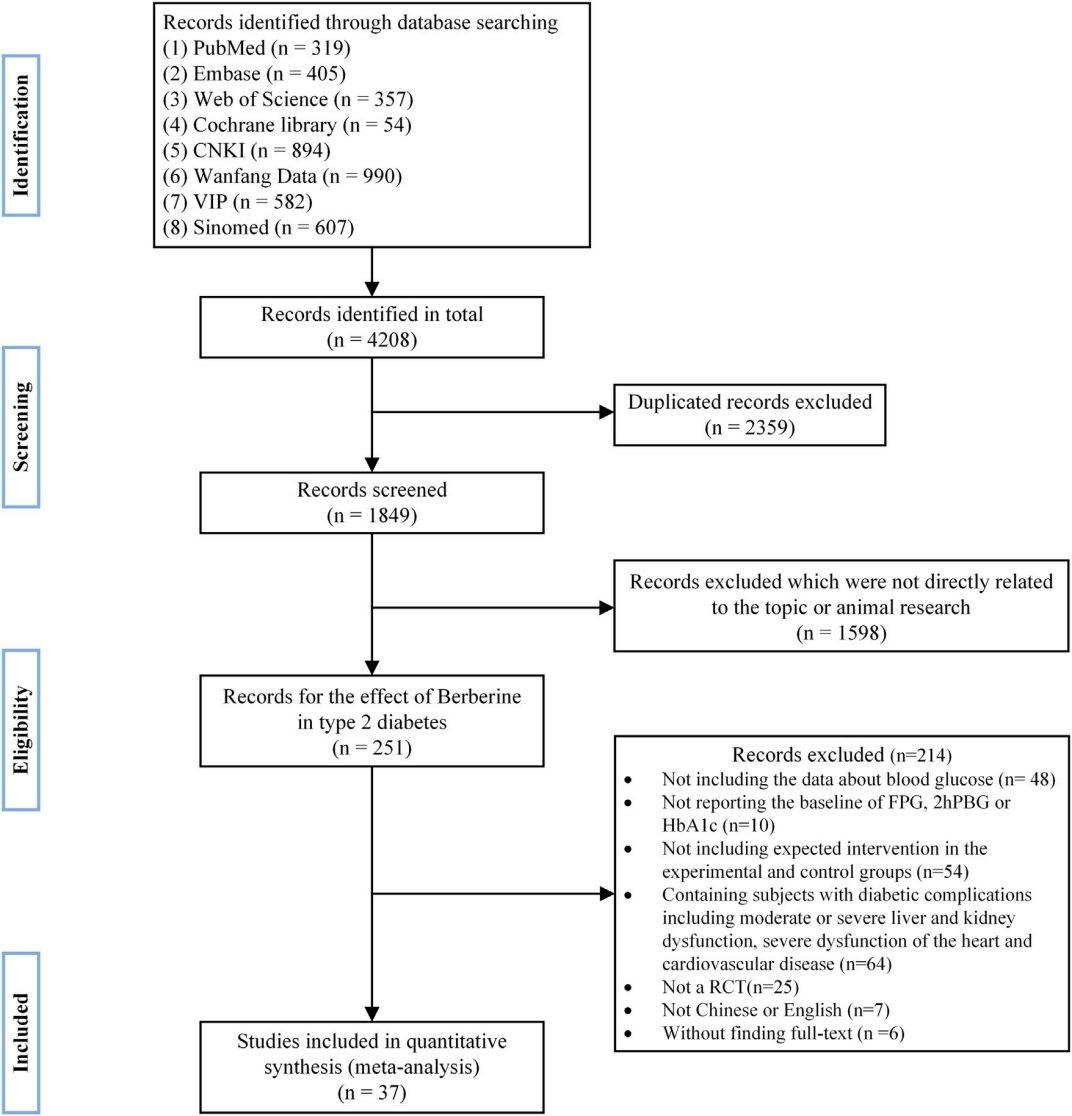
Flow diagram.

A total of 4,208 publications were initially retrieved by literature search. After excluding duplicate references and carefully reading the titles, abstracts, and the full texts, 37 randomized controlled trials (Liu, 2004; Cao, 2007; Li and Liu, 2007; You and Liu, 2007; Li, 2008; Liu and Hu, 2008; Wang, 2008; Xu, 2008; Yang, 2008; Zhang et al., 2008; Sheng, 2009; Zhu et al., 2009; Gu et al., 2010; Yang, 2010; Ye, 2010; Xiang et al., 2011; Yin, 2011; Xue et al., 2012; Zhang and Yuan, 2012; Liu, 2013; Li, 2014; Yao and Xie, 2015; Zhu et al., 2015; Lang and Zhu, 2016; Li, 2016; Dong et al., 2017; Sun, 2017; Zhang, 2017; Zhang and Chen, 2017; Fan et al., 2018; Rashidi et al., 2018; Jiang and Wang, 2019; Yang et al., 2020; Yu, 2020; Zhu et al., 2020; Wei and Deng, 2021; Ye, 2021) were finally included in the quantitative meta-analysis. Detailed retrieval steps were shown in Figure 1.

### 3.2 Description of included studies

After systematic selection and evaluation, 37 articles were finally included, as shown in Table 2. Three articles (Zhang et al., 2008; Gu et al., 2010; Rashidi et al., 2018) were English studies, while the rest were published in Chinese, all of which were journal articles. Of these studies, one (Rashidi et al., 2018) was conducted in Iran and the others were carried out in China, enrolling 3,048 patients with T2DM in total. Additionally, all studies were RCTs: 27 studies were conducted in a dual-arm parallel group design, and the other ten studies were divided into three groups.

**TABLE 2:** Characteristics of included studies in the meta-analysis.

**Study**	**Year**	**Region**	**Drug**	**Dose (mg, min-max/d)**		**Lifestyle Intervention**	**Mean Age (SD)**	**Size**	**Length of Trial**	**Efficacy**
Li	2012	China	BBR	1500	tid	Yes	45.5 (2.5)	60	6m	FPG, 2hPBG, HbA1c
			no durg	\	\			60		
Wang	2008	China	BBR	900	tid	Yes	47.74 (7.39)	31	12w	FPG, 2hPBG, HbA1c
			no durg	\	\		46.87 (7.74)	30		
Lang	2016	China	BBR	1500	tid	Yes	46.3 (4.7)	50	3m	FPG, 2hPBG, HbA1c
			placebo	\	\					
Xiang	2011	China	BBR	1200	tid	Yes	Unknown	20	12w	FPG, 2hPBG, HbA1c
			placebo	\	\			20		
Gu and Zhang	2010	China	BBR	1000	qd	Unknown	51 (9)	30	3m	FPG, 2hPBG, HbA1c
			placebo	\	\		50 (10)	30		
Rashidi	2018	Iran	BBR	1000	bid	Yes	50.18 (4.22)	42	4w	FPG, 2hPBG
			placebo	\	\		45.12 (9.55)	42		
Zhang and Li	2008	China	BBR	1000	bid	Unknown	51 (10)	57	3m	FPG, 2hPBG, HbA1c
			placebo	\	\		Unknown	49		
You and Liu	2007	China	OHAs^**1**^	\	\	Yes	Unknown	19	3w	FPG
			OHAs+BBR	\+900	\ /tid			35		
Zhu and Jiang	2020	China	OHAs^**2**^	1500	tid	Unknown	58.80 (12.27)	25	12w	FPG, HbA1c
			OHAs+BBR	\	\		60.46 (11.73)	25		
Liu	2008	China	MET	1500	tid	Yes	53.07 (8.51)	30	8w	FPG, 2hPBG, HbA1c
			MET+BBR	1500 + (900∼1500)	tid/tid		52.00 (9.81)	30		
Sun	2017	China	MET	1500	tid	Yes	58.34 (11.21)	91	8w	FPG, 2hPBG, HbA1c
			MET+BBR	1500 + 90	tid/tid		58.95 (10.57)	91		
Liu	2004	China	MET	1500	tid	Yes	55.2 (3.6)	33	14d	FPG, 2hPBG
			MET+BBR	(750∼1500)+1500	tid/tid		53.2 (2.8)	35		
Dong	2017	China	MET	1500	tid	Yes	51.34 (4.43)	49	12w	FPG, 2hPBG, HbA1c
			MET+BBR	1500 + 900	tid/tid		52.23 (4.41)	49		
Fan	2018	China	MET	1500	tid	Yes	52.71 (7.89)	40	3m	FPG, 2hPBG, HbA1c
			MET+BBR	1500 + 1500	tid/tid		53.27 (8.15)	40		
Liu	2013	China	MET	1000∼2000	tid	Yes	47.5 (6.5)	32	16w	FPG, 2hPBG, HbA1c
			MET+BBR	(1000∼2000) + 1500	tid/tid		46.5 (7.2)	36		
Jiang	2019	China	MET	1500	tid	Yes	62.76 (4.59)	51	12w	FPG, 2hPBG, HbA1c
			MET+BBR	1500 + 900	tid/tid		63.19 (4.82)	51		
Zhang and Yuan	2012	China	MET	1500	tid	Yes	Unknown	38	3m	FPG, 2hPBG, HbA1c
			MET+BBR	1500 + (1500∼2400)	tid/tid			38		
Yin	2011	China	MET	1500	tid	Yes	Unknown	30	6m	FPG, 2hPBG, HbA1c
			MET+BBR	1500 + 1500	tid/tid			30		
Yang	2020	China	MET	2000	bid	Yes	49.7 (7.4)	96	3m	FPG, 2hPBG, HbA1c
			MET+BBR	2000 + 1500	bid/tid		49.9 (7.8)	96		
Yu	2020	China	MET+placebo	1000∼2000	qd	Yes	42.58 (6.44)	52	24w	FPG, 2hPBG, HbA1c
			MET+BBR	(1000∼2000) + 1500	qd/tid		43.00 (8.35)	56		
Yang	2008	China	MET	750∼1500	tid	Yes	53.6 (12.9)	30	3m	FPG, 2hPBG, HbA1c
			BBR	1500	tid		55.3 (11.5)	30		
			no drug	\	\		55.4 (10.7)	30		
Cao	2007	China	MET	1500	tid	Yes	53.6 (12.9)	30	3m	FPG, 2hPBG, HbA1c
			BBR	1500	tid		55.3 (11.5)	30		
			no drug	\	\		55.4 (10.7)	30		
Li	2008	China	MET	1500	tid	Yes	61 (12)	17	12w	FPG, 2hPBG
			BBR	900	tid			17		
			MET+BBR	1500 + 900	tid/tid			18		
Li^a^	2016	China	MET	750	tid	Yes	55.98 (8.24)	30	12w	FPG, 2hPBG, HbA1c
			BBR	900	tid			30		
			MET+BBR	750 + 450	tid/tid			30		
Zhu^a^	2009	China	MET	150	tid	Yes	42 (12)	50	3m	FPG, 2hPBG, HbA1c
			BBR	1500	tid			55		
			MET+BBR	150 + 1500	tid/tid			55		
Xue^a^	2012	China	MET	750	tid	Yes	54 (11)	45	12w	FPG, 2hPBG, HbA1c
			BBR	900	tid			42		
			MET+BBR	750 + 450	tid/tid			44		
Zhang(a)^a^	2017	China	MET	750	qd	Yes	55.2 (8.1)	33	3m	FPG, 2hPBG, HbA1c
			BBR	9000	tid		54.9 (7.9)	33		
			MET+BBR	750 + 9000	qd/tid		56.3 (5.7)	33		
Ye^a^	2021	China	MET	750	qd	Unknown	65.23 (1.86)	30	180d	FPG, 2hPBG, HbA1c
			BBR	9000	tid		65.39 (1.36)	30		
			MET+BBR	750 + 9000	qd/tid		65.97 (1.76)	30		
Zhang(b)^a^	2017	China	MET	750	qd	Yes	58.24 (6.15)	40	3m	FPG, 2hPBG, HbA1c
			BBR	9000	tid		58.13 (6.24)	40		
			MET+BBR	750 + 9000	qd/tid		58.91 (6.58)	40		
Zhu	2015	China	GLC	30	qd	Yes	65.6 (7.2)	59	12w	FPG, 2hPBG, HbA1c
			GLC+BBR	30 + 300	qd/tid		66.4 (7.6)	59		
Li and Liu	2007	China	GLP	15	qd	Unknown	52 (14)	50	60d	FPG, 2hPBG, HbA1c
			BBR	900	tid			51		
			GLP +BBR	15 + 900	qd/tid			51		
Yao	2015	China	GLP+MET	5 + 1500	qd/tid	Yes	43.2 (3.2)	38	3m	FPG, 2hPBG, HbA1c
			GLP+MET+BBR	5 + 1500 + 1500	qd/tid/tid		44.9 (2.8)	38		
Sheng	2009	China	GLP+MET	10 + 1500	bid/tid	Yes	51.9 (10.7)	30	3m	FPG
			GLP+MET+BBR	10 + 1500 + 1500	bid/tid/tid		48.0 (10.2)	30		
Ye	2010	China	GLM+MET	200 + 1500	bid/tid	Unknown	42.5 (8.6)	40	3m	FPG, 2hPBG, HbA1c
			GLM+MET+BBR	200 + 1500 + 1500	bid/tid/tid		43.5 (9.8)	40		
Xu and Yu	2008	China	PIO	30	qd	Yes	43.4 (2.1)	32	12w	FPG, 2hPBG
			PIO+BBR	30 + 900	qd/tid			32		
Wei	2021	China	MET+Ac	1000 + 150	bid/tid	Unknown	51.6 (7.2)	58	3m	FPG, 2hPBG, HbA1c
			MET+BBR+Ac	1000 + 1500 + 150	bid/tid/tid		50.8 (6.9)	58		
Yang	2010	China	Ac	75∼300	tid	Yes	53.9 (9.8)	25	3m	FPG, 2hPBG, HbA1c
			Ac+BBR	(75∼300 )+ 2400	tid/tid		55.5 (9.1)	24		

MET metformin, BBR Berberine, SUS Sulfonylureas, GLP glipizide, GLC gliclazide, GLM glimepiride, TZDs thiazolidinediones, PIO pioglitazone, Ac acarbose.

^a^ : The Sulfonylureas originally taken were also used during the interventions, but the specific dose wasn’t mentioned.

1: The types and doses of OHAs are not mentioned.

2: The types of OHAs included PIO (10/25), GLM (10/25), GLC (5/25). The dose of OHAs was not mentioned.

Of these 37 studies, seven studies (Wang, 2008; Zhang et al., 2008; Gu et al., 2010; Xiang et al., 2011; Li, 2014; Lang and Zhu, 2016; Rashidi et al., 2018) compared BBR with placebo or no drug, while 20 studies (Liu, 2004; You and Liu, 2007; Liu and Hu, 2008; Xu, 2008; Sheng, 2009; Yang, 2010; Ye, 2010; Yin, 2011; Zhang and Yuan, 2012; Liu, 2013; Yao and Xie, 2015; Zhu et al., 2015; Dong et al., 2017; Sun, 2017; Fan et al., 2018; Jiang and Wang, 2019; Yang et al., 2020; Yu, 2020; Zhu et al., 2020; Wei and Deng, 2021) made a comparison between a co-intervention of BBR and one or two types of OHAs and a control of the same OHAs. In addition, Ten studies (Cao, 2007; Li and Liu, 2007; Li, 2008; Yang, 2008; Zhu et al., 2009; Xue et al., 2012; Li, 2016; Zhang, 2017; Zhang and Chen, 2017; Ye, 2021) made comparisons among a co-intervention of BBR and one type of OHAs, a control of the same OHAs, a control of BBR or no drug. Among these 37 studies, five studies (Zhu et al., 2009; Xue et al., 2012; Zhang, 2017; Zhang and Chen, 2017; Ye, 2021) retained the original dosage of sulfonylureas but did not specify dose. A study (You and Liu, 2007) did not explain the types and doses of OHAs. Another study (Zhu et al., 2020) did not specify the doses of OHAs. The OHAs used in the control group included metformin, sulfonylureas (including glipizide, gliclazide, and glimepiride), thiazolidinediones (including pioglitazone), and acarbose.

Study periods ranged anywhere from 14 days to 6 months, with three trials of 180 days, 16 trials lasted for 90 days (3m), ten trials lasted for 84 days (12w), and five trials lasted for 21-60 days (3w-2m). A trial (Liu, 2004) lasted for 14 days (2w), and another trial (Liu, 2013) lasted for 112 days (16w).

The most common dose of BBR ranged 0.9–2.4 g/d. In two trials (Yang, 2010; Zhang and Yuan, 2012), the dosage of BBR ranged from 1.5 to 2.4 g/d; in three trials (Zhang, 2017; Zhang and Chen, 2017; Ye, 2021), patients received 9.0 g/d; in a study (Sun, 2017), patients received 0.09 g/d; in a study (Zhu et al., 2015), patients received 0.3 g/d; and in the remaining 30 trials, the dose of BBR ranged 0.9–1.5 g/d.

In Table 3 and 4, according to the inclusion criteria, the control group included other OHAs, placebo, or lifestyle intervention group, while the experimental group included BBR or the combined use of BBR and the interventions in the control group. For example, in a study (Li, 2014), the placebo group was the control group and the BBR group was the experimental group; on the other hand, in a study published by Li in 2008 (Li, 2008), the metformin group was the control group and the BBR combined with metformin group was the experimental group. Table 3 was organized according to baseline mean FPG and Table 4 ranked according to baseline mean HbA1c (the mean was calculated as the sum of the control group and experimental group divided by two). The mean of FPG was included in each of the 37 trials, and four subgroups were divided according to baseline mean FPG: less than 7.5 mmol/L, 7.5–8.8 mmol/L, 8.8–10.0 mmol/L, and greater than or equal to 10.0 mmol/L; The mean of HbA1c was included in 31 of 37 trials, and two subgroups were divided according to baseline mean HbA1c: less than 9.0% and greater than or equal to 9.0%. Detailed contents were shown in Table 3 and Table 4.

**TABLE 3:** Baseline mean FPG of included studies in the meta-analysis.

**study**	**Baseline FPG**		
	**control**	**experimental**	**mean**
**Baseline mean FPG < 7.5 mmol/L**			
Gu et al. (2010)	6.9	6.9	6.9
Zhang et al. (2008)	6.8	7.0	6.9
Yin (2011)	7.4	7.2	7.3
Yao and Xie (2015)	7.5	7.3	7.4
Xu (2008)	7.4	7.4	7.4
Wang (2008)	7.46	7.37	7.415
**Baseline mean FPG: 7.5 ∼ 8.8 mmol/L**			
You and Liu (2007)	7.9	7.4	7.65
Ye (2010)	7.60	7.80	7.7
Yang (2008)	7.98	8.28	8.13
Yu (2020)	8.21	8.17	8.19
Cao (2007)	8.06	8.37	8.215
Xiang et al. (2011)	8.26	8.37	8.315
Sheng (2009)	8.56	8.61	8.585
**Baseline mean FPG: 8.8 ∼ 10.0 mmol/L**			
Liu (2013)	8.7	9.0	8.85
Sun (2017)	8.75	8.96	8.855
Zhu et al. (2020)	8.93	8.96	8.945
Liu and Hu (2008)	9.03	9.01	9.02
Dong et al. (2017)	9.15	9.14	9.145
Wei and Deng (2021)	9.2	9.2	9.2
Liu (2004)	9.4	9.1	9.25
Yang et al. (2020)	9.2	9.3	9.25
Li (2014)	9.31	9.32	9.315
Lang and Zhu (2016)	9.33	9.35	9.34
Fan et al. (2018)	9.59	9.60	9.595
Jiang and Wang (2019)	10.01	9.79	9.9
Zhang and Yuan (2012)	10.01	9.82	9.915
**Baseline mean FPG**≥ **10.0 mmol/L**			
Zhu et al. (2015)	10.23	9.93	10.08
Rashidi et al. (2018)	9.93	10.67	10.3
Yang (2010)	10.5	10.7	10.6
Li (2008)	10.7	10.6	10.65
Zhang (2017)	10.9	11.0	10.95
Li and Liu (2007)	11.2	11.1	11.15
Li (2016)	11.1	11.2	11.15
Xue et al. (2012)	11.0	11.3	11.15
Ye (2021)	10.75	11.97	11.36
Zhang and Chen (2017)	11.4	11.5	11.45
Zhu et al. (2009)	13.3	13.3	13.3

FPG, fasting plasma glucose.

**TABLE 4:** Baseline mean HbA1c of included studies in the meta-analysis.

**study**	**Baseline HbA1c**		
	**control**	**experimental**	**mean**
**Baseline mean HbA1c < 9.0%**			
Wang (2008)	7.33	7.10	7.22
Yin (2011)	7.2	7.4	7.30
Yao and Xie (2015)	7.6	7.3	7.45
Cao (2007)	7.50	7.47	7.49
Zhang et al. (2008)	7.6	7.5	7.55
Yang (2008)	7.52	7.58	7.55
Ye (2010)	7.40	7.70	7.55
Gu et al. (2010)	7.7	7.6	7.65
Yu (2020)	7.71	7.67	7.69
Zhang and Yuan (2012)	8.01	8.15	8.08
Liu and Hu (2008)	8.12	8.08	8.10
Yang (2010)	8.30	8.20	8.25
Li (2014)	8.49	8.51	8.50
Lang and Zhu (2016)	8.53	8.52	8.53
Li and Liu (2007)	8.4	8.8	8.60
Zhu et al. (2015)	8.67	8.84	8.76
Sun (2017)	8.75	8.81	8.78
Liu (2013)	8.7	9.0	8.85
Dong et al. (2017)	8.85	8.86	8.86
Xiang et al. (2011)	8.92	8.97	8.95
Fan et al. (2018)	9.04	8.93	8.99
**Baseline mean HbA1c ≥ 9.0%**			
Yang et al. (2020)	9.1	9.2	9.15
Wei and Deng (2021)	9.1	9.3	9.20
Jiang and Wang (2019)	9.28	9.21	9.25
Zhu et al. (2020)	9.25	9.29	9.27
Li (2016)	9.2	9.4	9.30
Zhang and Chen (2017)	9.2	9.4	9.30
Xue et al. (2012)	9.3	9.5	9.40
Zhang (2017)	9.5	9.6	9.55
Ye (2021)	9.63	9.64	9.64
Zhu et al. (2009)	10.9	10.9	10.90
Xu (2008)	Unknown		
You and Liu (2007)	Unknown		
Sheng (2009)	Unknown		
Liu (2004)	Unknown		
Rashidi et al. (2018)	Unknown		
Li (2008)	Unknown		

HbA1c, glycosylated hemoglobin.

### 3.3 Risk of bias

**FIGURE 2:**
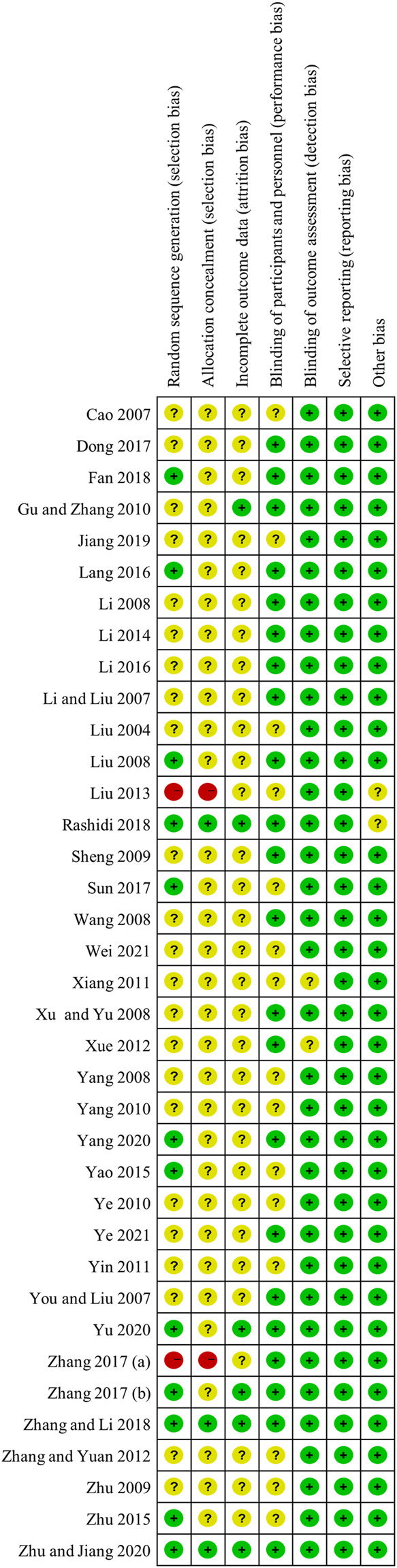
Risk of bias of assessment.

Most studies included in the meta-analysis was of moderate quality, mainly due to poor reporting of allocation concealment and blinding of participants and personnel. Among the 37 RCTs included in the meta-analysis, 12 studies provided definite information on random sequence generation. Three trials described allocation concealment, and six trials exhibited blinding of participants and personnel. In addition, incomplete outcome data or selective reporting was perceived as low risk nearly in all included studies, and risk of other bias was assessed as low mostly. The detailed quality assessments across the recruited studies were presented in Figure 2.

### 3.4 Main results of the meta-analysis

#### 3.4.1 FPG

FPG was used to compare the efficacy of the experimental group with the control group in 37 studies (Liu, 2004; Cao, 2007; Li and Liu, 2007; You and Liu, 2007; Li, 2008; Liu and Hu, 2008; Wang, 2008; Xu, 2008; Yang, 2008; Zhang et al., 2008; Sheng, 2009; Zhu et al., 2009; Gu et al., 2010; Yang, 2010; Ye, 2010; Xiang et al., 2011; Yin, 2011; Xue et al., 2012; Zhang and Yuan, 2012; Liu, 2013; Li, 2014; Yao and Xie, 2015; Zhu et al., 2015; Lang and Zhu, 2016; Li, 2016; Dong et al., 2017; Sun, 2017; Zhang, 2017; Zhang and Chen, 2017; Fan et al., 2018; Rashidi et al., 2018; Jiang and Wang, 2019; Yang et al., 2020; Yu, 2020; Zhu et al., 2020; Wei and Deng, 2021; Ye, 2021) involving 3,048 patients. The meta-analysis showed that, after treatment, FPG in the experimental group was lower than that in the control group (random effects model, WMD = -0.82 mmol/L, 95% CI (-0.95, -0.70), *p* < 0.001). This result was statistically significant. However, there was significant heterogeneity (I^2^ = 60% > 50%, *p* < 0.001). Therefore, we performed a subgroup analysis according to baseline mean FPG.

**FIGURE 3:**
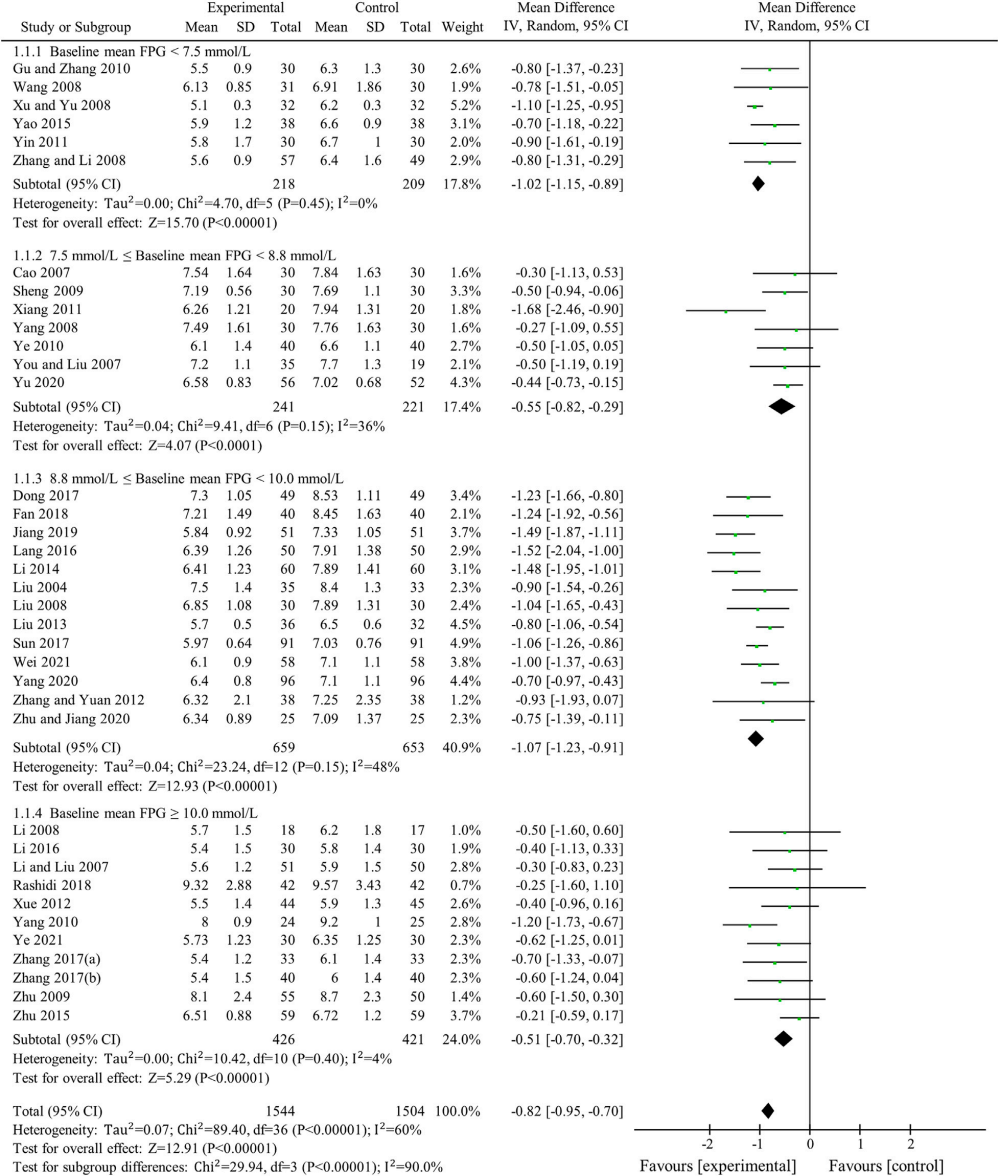
Meta-analysis of the effect of BBR on FPG.

As demonstrated by the result of subgroup analysis in Table 5 and Figure 3, FPG in the experimental group was lower than that in the control group after treatment in each subgroup. The result was statistically significant (*p* < 0.001) without significant heterogeneity (I^2^ < 50%).

**TABLE 5:** The results of subgroup analyses.

Subgroups	n	WMD	95% Cl	*p*-value	Heterogeneity between studies
FPG (mmol/L)
Overall	37	-0.82	(-0.95, -0.70)	<0.001	I^2^ = 60% *p* < 0.001
Baseline mean FPG
<7.5 mmol/L	6	-1.02	(-1.15, -0.89)	<0.001	I^2^ = 0% *p* = 0.45
7.5 mmol/L ∼ 8.8 mmol/L	7	-0.55	(-0.82, -0.29)	<0.001	I^2^ = 36% *p* = 0.15
8.8 mmol/L ∼ 10.0 mmol/L	13	-1.07	(-1.23, -0.91)	<0.001	I^2^ = 48% *p* = 0.03
≥10.0 mmol/L	11	-0.51	(-0.70, -0.32)	<0.001	I^2^ = 4% *p* = 0.40
HbA1c (%)
Overall	31	-0.63	(-0.72, -0.53)	<0.001	I^2^ = 52% *p* < 0.001
Baseline mean HbA1c
<9.0%	21	-0.58	(-0.69, -0.46)	<0.001	I^2^ = 49% *p* = 0.006
≥9.0%	10	-0.79	(-0.90, -0.67)	<0.001	I^2^ = 6% *p* = 0.38
Baseline mean FPG					
<7.5 mmol/L	5	-0.60	(-0.71, -0.49)	<0.001	I^2^ = 0% *p* = 0.92
7.5 mmol/L ∼ 8.8 mmol/L	5	-0.57	(-0.98, -0.17)	0.006	I^2^ = 62% *p* = 0.03
8.8 mmol/L ∼ 10.0 mmol/L	12	-0.72	(-0.87, -0.57)	<0.001	I^2^ = 62% *p* = 0.002
≥10.0 mmol/L	9	-0.51	(-0.70, -0.33)	<0.001	I^2^ = 4% *p* = 0.41
2hPBG (mmol/L)
Overall	34	-1.16	(-1.36, -0.96)	<0.001	I^2^ = 68% *p* < 0.001
Baseline mean HbA1c
<9.0%	21	-1.38	(-1.69, -1.08)	<0.001	I^2^ = 71% *p* < 0.001
≥9.0%	9	-0.93	(-1.21, -0.64)	<0.001	I^2^ = 50% *p* = 0.04
Lacking data of baseline mean HbA1c	4	-0.78	(-0.93, -0.63)	<0.001	I^2^ = 0% *p* = 0.72
Baseline mean FPG					
<7.5 mmol/L	6	-1.35	(-1.97, -0.73)	<0.001	I^2^ = 72% *p* = 0.003
7.5 mmol/L ∼ 8.8 mmol/L	5	-1.21	(-1.85, -0.58)	<0.001	I^2^ = 61% *p* = 0.04
8.8 mmol/L ∼ 10.0 mmol/L	12	-1.19	(-1.58, -0.79)	<0.001	I^2^ = 83% *p* < 0.001
≥10.0 mmol/L	11	-1.10	(-1.34, -0.86)	<0.001	I^2^ = 0% *p* = 0.85

CI, confidence interval; WMD, weighted mean differences, HbA1c glycosylated hemoglobin, FPG, fasting plasma glucose, 2hPBG, 2-h postprandial blood glucose.

**FIGURE 4:**
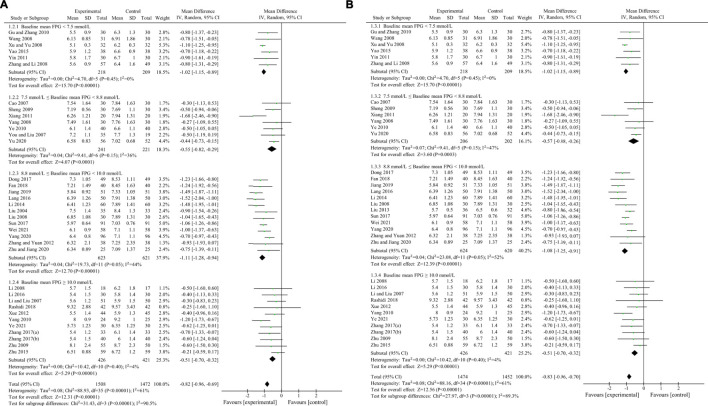
Sensitivity analyses by exclusion of apparently poor quality reports and exclusion of studies with too short treatment duration (≤3w). **(A)** Sensitivity analysis by exclusion of apparently poor quality reports **(B)** Sensitivity analysis by exclusion of studies with too short treatment duration (≤3w).

**FIGURE 5:**
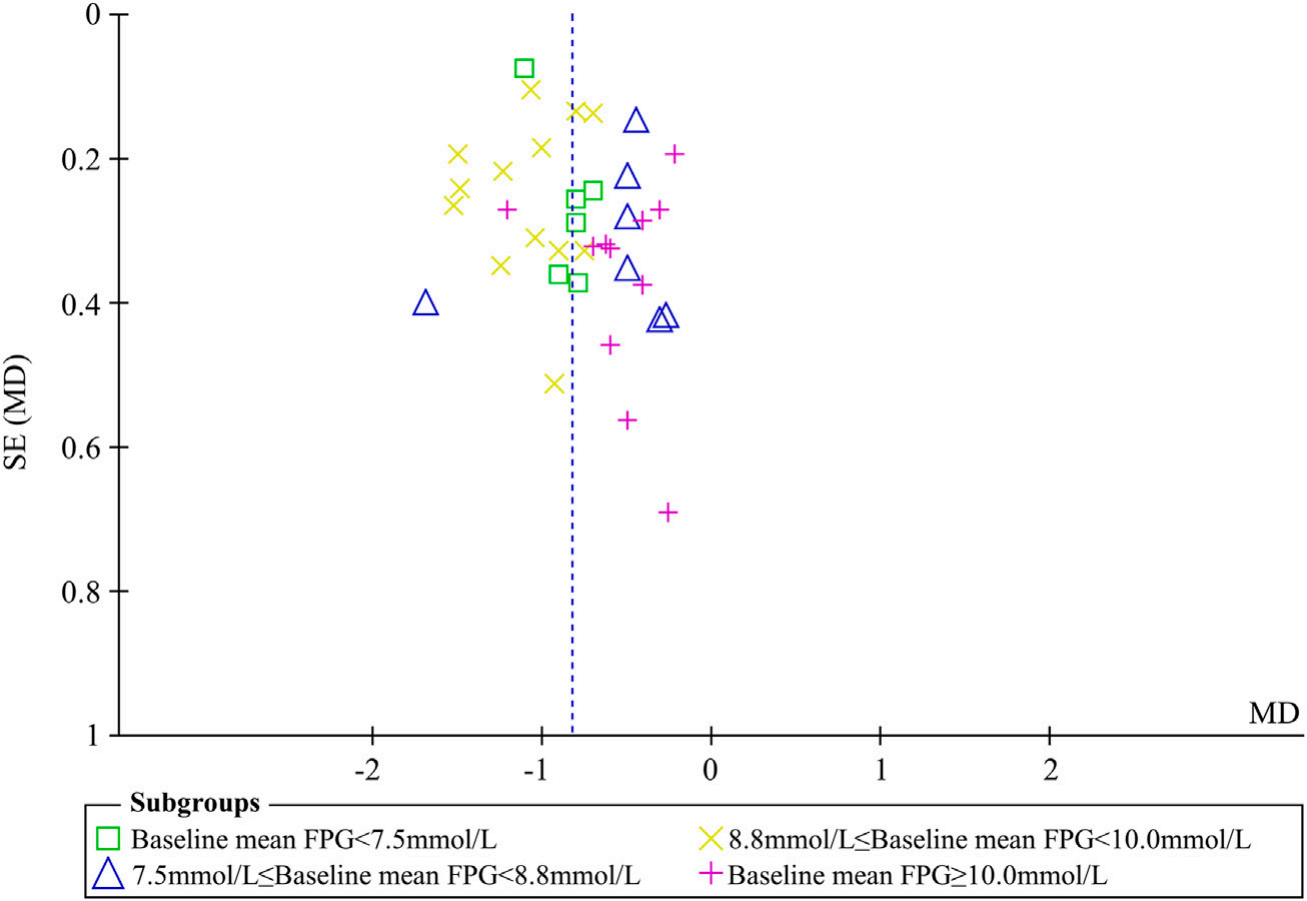
Funnel plot for FPG.

Sensitivity analyses were conducted to assess changes in FPG by excluding the report of apparently poor quality (Liu, 2013) and eliminating studies with too short treatment duration (≤3w) (Liu, 2004; You and Liu, 2007). The results of meta-analysis were not substantially changed (Excluding the report of apparently poor quality: WMD = -0.82 mmol/L, 95% CI (-0.96, -0.69), *p* < 0.001, I^2^ = 61%; Eliminating studies with too short treatment duration: WMD = -0.83 mmol/L, 95% CI (-0.96, -0.70), *p* < 0.001, I^2^ = 61%) (Figures 4A,B). Egger’s test showed that there was no obvious publication bias (*p* = 0.079 > 0.05) and the funnel plots for FPG was largely symmetric (Figure 5).

#### 3.4.2 HbA1c

**FIGURE 6:**
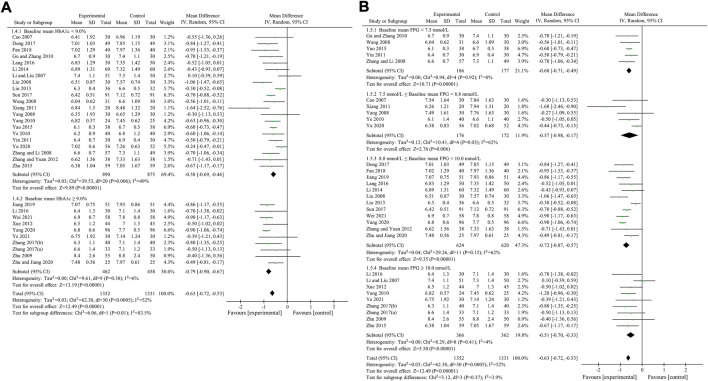
Meta-analysis of the effect of BBR on HbA1c. **(A)** Subgroup analysis according to baseline mean HbA1c. **(B)** Subgroup analysis according to baseline mean FPG.

HbA1c was used to compare the efficacy of the experimental group with the control group in 31 studies (Cao, 2007; Li and Liu, 2007; Liu and Hu, 2008; Wang, 2008; Yang, 2008; Zhang et al., 2008; Zhu et al., 2009; Gu et al., 2010; Yang, 2010; Ye, 2010; Xiang et al., 2011; Yin, 2011; Xue et al., 2012; Zhang and Yuan, 2012; Liu, 2013; Li, 2014; Yao and Xie, 2015; Zhu et al., 2015; Lang and Zhu, 2016; Li, 2016; Dong et al., 2017; Sun, 2017; Zhang, 2017; Zhang and Chen, 2017; Fan et al., 2018; Jiang and Wang, 2019; Yang et al., 2020; Yu, 2020; Zhu et al., 2020; Wei and Deng, 2021; Ye, 2021) involving 2,683 patients. The meta-analysis showed that, after treatment, HbA1c in the experimental group was lower than that in the control group (random effects model, WMD = -0.63%, 95% CI (-0.72, -0.53), *p* < 0.001). This result was statistically significant. However, there was significant heterogeneity (I^2^ = 52% > 50%, *p* < 0.001). Therefore, we performed subgroup analyses according to baseline mean HbA1c and FPG. The result of subgroup analysis is presented in Table 5 and Figure 6. When subgroup analysis was performed according to baseline mean HbA1c, after treatment, in each subgroup, HbA1c in the experimental group was lower than that in the control group. The result was statistically significant (*p* < 0.001) without significant heterogeneity (I^2^ < 50%) (Figure 6A). When subgroup analysis was performed according to baseline mean FPG, after treatment, HbA1c in the experimental group was lower than that in the control group and the result was statistically significant (*p* < 0.05) in each subgroup. When baseline mean FPG was <7.5 or ≥10.0 mmol/L, there was no significant heterogeneity (I^2^ < 50%, *p* > 0.1). However, when baseline mean FPG was ≥7.5 and <10.0 mmol/L, there was significant heterogeneity (I^2^ > 50%) (Figure 6B).

Egger’s test showed that there was no obvious publication bias (*p* = 0.812 > 0.05).

#### 3.4.3 2hPBG

**FIGURE 7:**
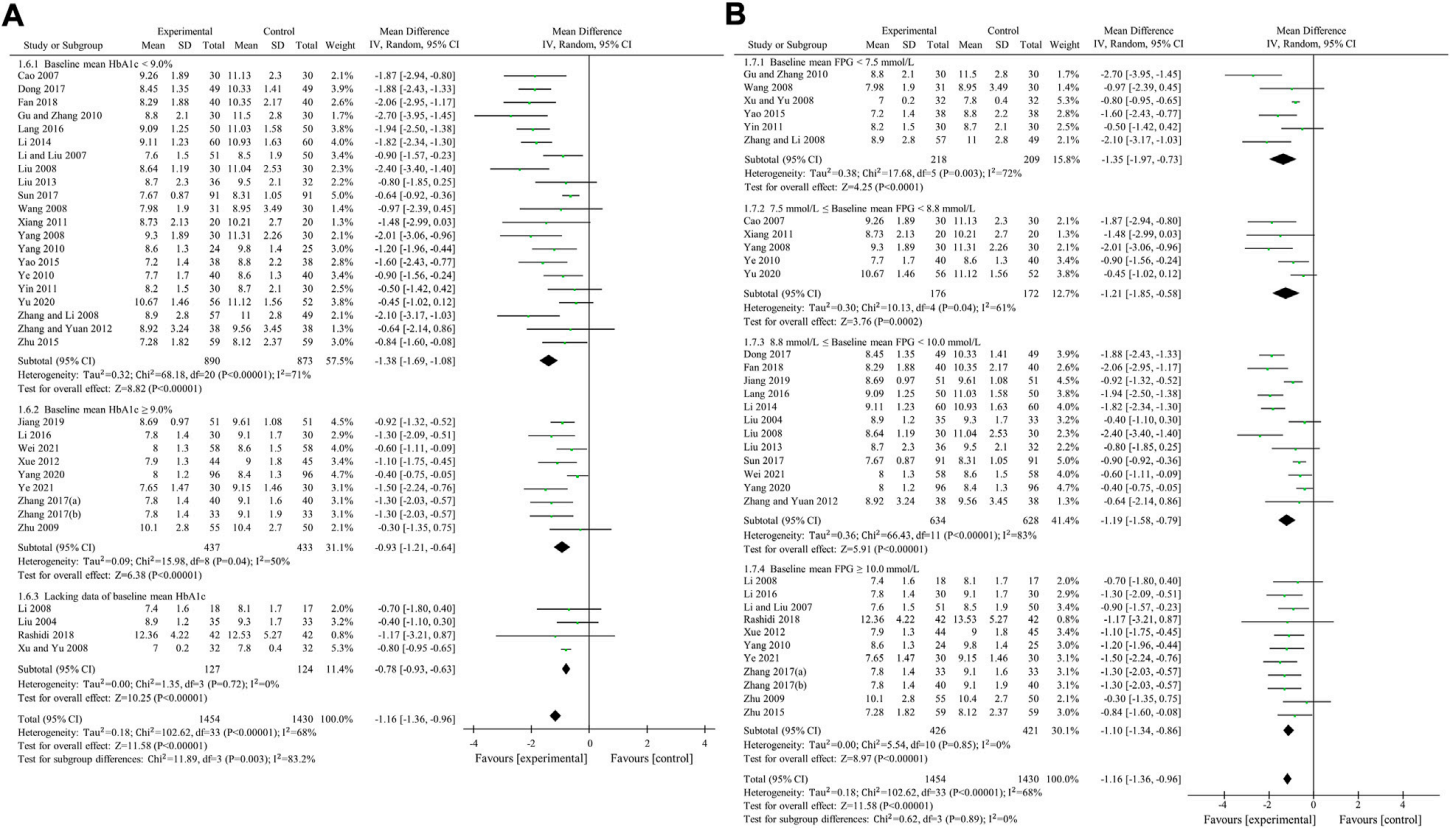
Meta-analysis of the effect of BBR on 2hPBG **(A)** Subgroup analysis according to baseline mean HbA1c **(B)** Subgroup analysis according to baseline mean FPG.

2hPBG was used to compare the efficacy of the experimental group with the control group in 34 studies (Liu, 2004; Cao, 2007; Li and Liu, 2007; Li, 2008; Liu and Hu, 2008; Wang, 2008; Xu, 2008; Yang, 2008; Zhang et al., 2008; Zhu et al., 2009; Gu et al., 2010; Yang, 2010; Ye, 2010; Xiang et al., 2011; Yin, 2011; Xue et al., 2012; Zhang and Yuan, 2012; Liu, 2013; Li, 2014; Yao and Xie, 2015; Zhu et al., 2015; Lang and Zhu, 2016; Li, 2016; Dong et al., 2017; Sun, 2017; Zhang, 2017; Zhang and Chen, 2017; Fan et al., 2018; Rashidi et al., 2018; Jiang and Wang, 2019; Yang et al., 2020; Yu, 2020; Wei and Deng, 2021; Ye, 2021) involving 2,884 patients. The meta-analysis showed that, after treatment, 2hPBG in the experimental group was lower than that in the control group (random effects model, WMD = -1.16 mmol/L, 95% CI (-1.36, -0.96), *p* < 0.001). This result was statistically significant. Meanwhile, there was also significant heterogeneity (I^2^ = 68% > 50%, *p* < 0.001). Therefore, we performed subgroup analyses according to the baseline mean HbA1c and FPG. The result of the subgroup analysis is presented in Table 5 and Figure 7. No matter how subgroups were divided, after treatment, in each subgroup, HbA1c in the experimental group was always lower than that in the control group and the result was statistically significant (*p* < 0.001). There was no significant heterogeneity (I^2^ < 50%, *p* > 0.1) only if baseline mean HbA1c was ≥9% or baseline mean FPG was ≥10.0 mmol/L (Figures 7A,B).

Egger’s test showed that there was obvious publication bias (*p* = 0.006 < 0.05).

#### 3.4.4 Safety of berberine on patients with T2DM

**FIGURE 8:**
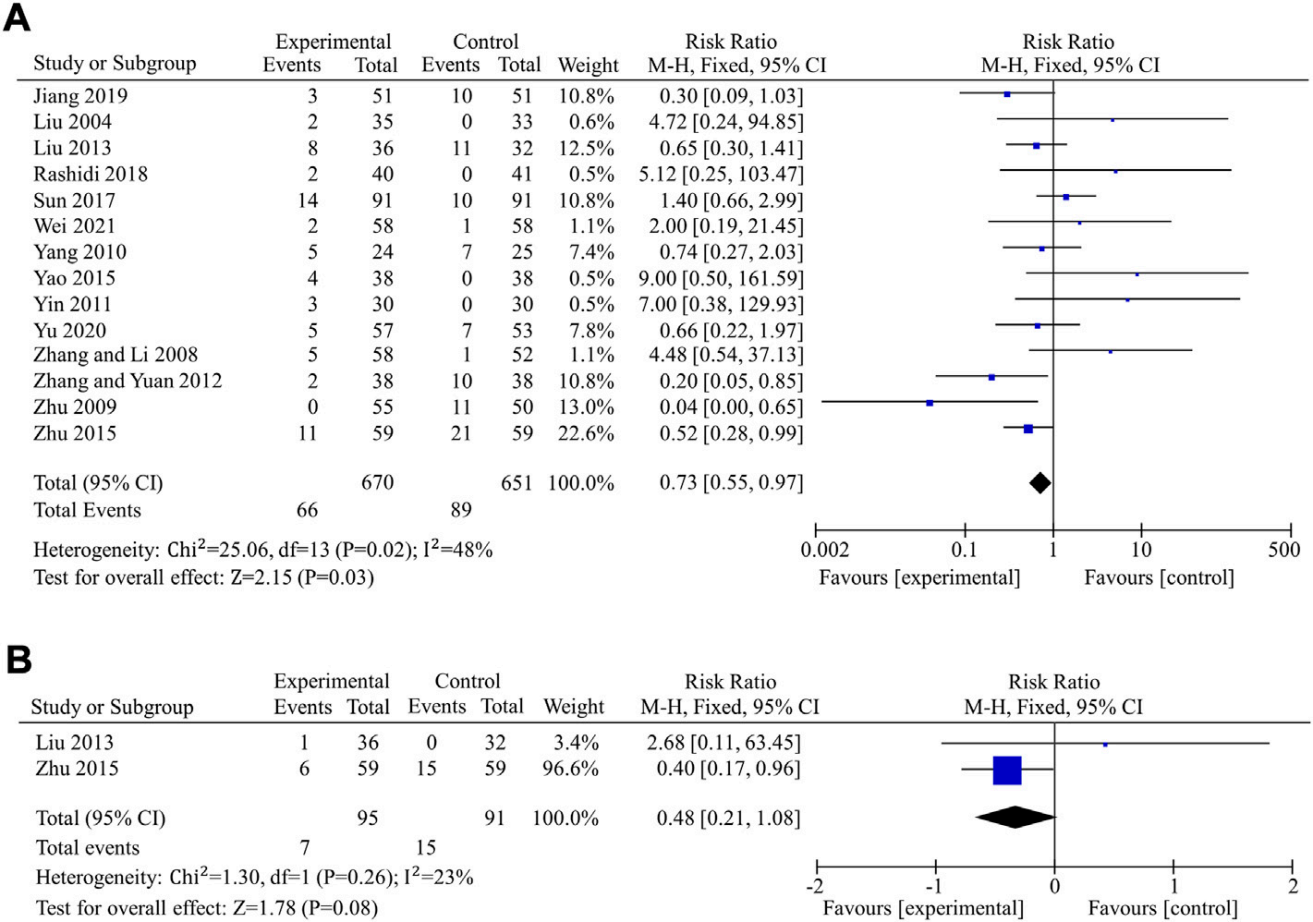
Meta-analysis of the effect of BBR on hypoglycemic events. **(A)** Total adverse events **(B)** Hypoglycemia.

The incidence of total adverse events was used to assess the safety of berberine in 14 studies (Liu, 2004; Zhang et al., 2008; Zhu et al., 2009; Yang, 2010; Yin, 2011; Zhang and Yuan, 2012; Liu, 2013; Yao and Xie, 2015; Zhu et al., 2015; Sun, 2017; Rashidi et al., 2018; Jiang and Wang, 2019; Yu, 2020; Wei and Deng, 2021), including a total of 670 patients in the experimental group and 651 in the control group. Data revealed that berberine alone or in combination with OHAs in the treatment of T2DM had a lower incidence of total adverse events and appeared to have better safety compared to the control group (fixed effects model, RR = 0.73, 95% CI (0.55, 0.97), *p* = 0.03). The result was of statistically significance without significant heterogeneity (I^2^ < 50%) (Figure 8A). Egger’s test showed that there was no obvious publication bias (*p* = 0.234 > 0.05).

Hypoglycemia was used to further compare the safety of the experimental group to the control group in nine studies (Li, 2008; Xu, 2008; Zhang et al., 2008; Sheng, 2009; Zhu et al., 2009; Liu, 2013; Yao and Xie, 2015; Zhu et al., 2015; Yu, 2020) involving 820 patients. In seven (Li, 2008; Xu, 2008; Zhang et al., 2008; Sheng, 2009; Zhu et al., 2009; Yao and Xie, 2015; Yu, 2020) of the nine studies, there were no hypoglycemic episodes in either the experimental group or the control group, and the remaining two studies (Liu, 2013; Zhu et al., 2015) reported hypoglycemia. Meta-analysis of those two studies, including 186 patients, showed that there were no significant differences in hypoglycemia between the experimental and control groups (fixed effects model, RR = 0.48, 95% CI (0.21, 1.08), *p* = 0.08). There was no significant heterogeneity (I^2^ = 23% < 50%, *p* > 0.1)) (Figure 8B).

#### 3.4.5 Certainty of evidences

The certainty of evidences of FPG, HbA1c and total adverse events was all moderate, because it was downgraded of a level for risk of bias. The certainty of evidence of 2hPBG was low because of risk of bias and publication bias. The certainty of evidence of hypoglycemia was very low because of risk of bias and imprecision (Table 6).

**TABLE 6:** Summary of findings table.

**Certainty assessment**							**No of patients**		**Effect**		**Certainty**	**Importance**
**No of studies**	**Study design**	**Risk of bias**	**Inconsistency**	**Indirectness**	**Imprecision**	**Other considerations**	**BBR+placebo/OHAs**	**placebo/OHAs**	**Relative (95% CI)**	**Absolute (95% CI)**		
FPG												
37	Randomized trials	serious^a^	not serious	not serious	not serious	none	1544	1504	-	MD 0.82 mmol/L lower(0.95 lower to 0.7 lower)	⊕⊕⊕○Moderate	IMPORTANT
HbA1c												
31	Randomized trials	serious^a^	not serious	not serious	not serious	none	1352	1331	-	MD 0.63 % lower(0.72 lower to 0.53 lower)	⊕⊕⊕○Moderate	IMPORTANT
2hPBG												
34	Randomized trials	serious^a^	not serious	not serious	not serious	publication bias strongly suspected^b^	1454	1430	-	MD 1.16 mmol/L lower (1.36 lower to 0.96 lower)	⊕⊕○○Low	IMPORTANT
Total adverse events												
14	Randomized trials	serious^a^	not serious	not serious	not serious	none	66/670 (9.9%)	89/651 (13.7%)	RR 0.73 (0.55 to 0.97)	37 fewer per 1,000 (from 62 fewer to 4 fewer)	⊕⊕⊕○Moderate	IMPORTANT
Hypoglycemia												
2	Randomized trials	serious^a^	not serious	not serious	very serious^c^	none	7/95 (7.4%)	15/91 (16.5%)	RR 0.48 (0.21 to 1.08)	86 fewer per 1,000(from 130 fewer to 13 more)	⊕○○○Very low	IMPORTANT

CI confidence interval, MD mean difference, RR risk ratio.

^a^ : Most information is from studies at low or unclear risk of bias. Potential limitations are likely to lower confidence in the estimate of effect.

^b^ : Egger's test showed that there was obvious publication bias.

^c^ : The optimal information size criterion is not met. The 95% CI overlaps no effect and fails to exclude important benefit.

## 4 Discussion

This meta-analysis showed that BBR was effective in the treatment of T2DM and reduced FPG, HbA1c, and 2hPBG in patients with T2DM. In subgroup analyses according to the baseline mean FPG and HbA1c, the reduction of FPG, HbA1c, or 2hPBG in each subgroup was statistically significant. Furthermore, we found that the baseline mean FPG was the source of heterogeneity of studies with FPG and both baseline mean FPG and HbA1c were sources of heterogeneity for HbA1c and 2hPBG, suggesting that the glucose-lowering effects of BBR correlated with baseline mean FPG and HbA1c. When baseline mean FPG was ≥10 mmol/L, I^2^ values of heterogeneity tests for studies focusing on FPG, HbA1c and 2hPBG were all less than 5%, indicating very small heterogeneity. So, the reduction effect of BBR on FPG, HbA1c, and 2hPBG was reliable in this subgroup. When baseline mean FPG was <7.5 mmol/L, I^2^ values of heterogeneity tests for studies focusing on FPG and HbA1c were both 0%, indicating that there is no significant heterogeneity. Therefore, the reduction effect of BBR on FPG and HbA1c was also reliable in this subgroup. Regarding safety, our findings suggested that berberine alone or in combination with OHAs in the treatment of T2DM did not increase the incidence of total adverse events and the risk of hypoglycemia. Berberine may even have the potential advantage of reducing the risk of adverse reactions when used as adjuvant therapy, but further studies are still needed to verify it. Therefore, berberine is a viable treatment option for T2DM. After rating with GRADE approach, the certainty of evidences of FPG, HbA1c and total adverse events was all moderate. It was low for 2hPBG and very low for hypoglycemia.

### 4.1 Comparison with previous meta-analysis

So far, there have been four meta-analyses on the therapeutic effects of BBR on T2DM. They have confirmed that BBR can improve blood glucose level, lipid metabolism, and inflammatory markers in diabetic patients, as well as treating hyperlipidemia and hypertension (Dong et al., 2012; Lan et al., 2015; Liang et al., 2019; Guo et al., 2021). The two latest meta-analyses also found that the effect of BBR in the treatment of diabetes was related to daily dosage, age, and treatment duration. But there was obvious heterogeneity among the included studies, especially in those focused on FPG. After subgroup analysis, the problem of heterogeneity still existed (Liang et al., 2019; Guo et al., 2021). Large heterogeneity reduced the reliability of the two meta-analyses. According to our meta-analysis, we found that one of the sources of heterogeneity of FPG was the baseline mean FPG, and sources of heterogeneity of HbA1c and 2hPBG included baseline mean FPG and HbA1c. In particular, for FPG, subgroup analysis based on baseline FPG reduced the heterogeneity of each subgroup to I^2^ < 50%. The reduction of heterogeneity made the conclusion of our meta-analysis more reliable.

### 4.2 Significant advantages and broad application prospects of BBR

Compared with existing glucose-lowering agents, berberine has significant advantages and broad application prospects, which are briefly reviewed as follows: 1) Berberine has multiple glucose-lowering mechanisms, including improving insulin resistance, regulating glucose metabolism, regulating blood lipid metabolism, anti-inflammatory effects, protecting islet cells, and antioxidant effects (Li et al., 2021). In addition, the latest research by Professor Yang Jinkui’s team in 2021 found that by inhibiting the KCNH6 potassium channel, berberine has a brand-new glucose-dependent insulinotropic effect and can treat diabetes while avoiding the risk of hypoglycemia (Zhao et al., 2021); 2) Berberine can relieve and prevent a variety of diabetic complications, including diabetic encephalopathy, diabetic nephropathy, diabetic cardiomyopathy, and has a protective effect on the nerves of diabetic peripheral neuropathy (Li et al., 2021); 3) One drug is commonly used for multiple purposes with a wide range of clinical values: berberine has multiple pharmacological effects, including anti-hyperglycemia, anti-hyperlipidemia, anti-hypertension, cardiovascular protection, anti-arrhythmia, improvement of congestive heart failure, anti-bacterial, fungal, viral and other pathogenic microorganisms, anti-inflammatory, antioxidant, anti-tumor and anti-platelet aggregation. Some pharmacodynamic effects of berberine have been reported in clinical application, but some pharmacological effects, including anti-tumor, are still limited to laboratory and studies (Li et al., 2008; Xing et al., 2017; Zhou et al., 2020); 4) Based on intestinal flora regulation, protection of the barrier of the intestinal mucosa and broad-spectrum antibacterial effects (Hou et al., 2022), berberine can treat diarrhea caused by a variety of bacteria, reduce the number of diarrhea (Lang and Zhu, 2016), while improving blood glucose abnormalities caused by intestinal microbial disorders (Zhu et al., 2020); 5) Berberine can inhibit genes for fat synthesis, inhibit the differentiation of preadipocytes into mature adipocytes, and reduce lipid accumulation, indicating that berberine can slightly reduce body weight (Li et al., 2008). In addition, berberine has been found to significantly reduce leptin levels in patients with type 2 diabetes (Wang, 2008), all of which suggest that berberine is suitable for the treatment of obese patients with type 2 diabetes; 6) Berberine, as a natural isoquinoline alkaloid extracted from Coptis chinensis and other plants, has the characteristics of low toxicity, fewer side effects, and good tolerance. Berberine has long been proved to be a drug without cytotoxic and mutagenic effects and with a very high safety factor through biochemical, pharmacological, and clinical studies. Reliable data suggests that berberine has fewer side effects than western medicine and no toxic effects on blood urea nitrogen and serum creatinine in most laboratory and clinical trials (Yang et al., 2020) (Chang et al., 2015; Liang et al., 2019) Only a small proportion of patients treated with berberine experience reflux, vomiting, diarrhea, or constipation; 7) Some marketed glucose-lowering agents are limited in their use due to their high price relative to the inexpensive berberine. Chinese medicinal materials are rich in resources, coptis chinensis is abundant, berberine is extracted from coptis chinensis, and the current drug production process has been artificially synthesized, so berberine is widely available and low-cost. In addition, berberine has fewer adverse reactions, easy to be accepted and adhered to by patients, and can also be used during pregnancy or delivery, which is worthy of clinical promotion (Liao and Hao, 2020).

Berberine can compensate for the weaknesses of existing glucose-lowering agents and play a good auxiliary role in the treatment of type 2 diabetes. Sulfonylurea hypoglycemic agents such as glipizide are mainly effective in patients with certain insulin synthesis and secretion function in islet β cells, and long-term use of sulfonylureas can cause a decrease in the number and affinity of sulfonylurea receptors on islet G cells, which disables the drug and also has the risk of leading to severe hypoglycemia. However, recent studies have shown that berberine can promote the regeneration of islet β cells to a certain extent and restore islet function. In addition, according to research findings, the blood glucose value of berberine combined with glipizide is more stably controlled than that of glipizide alone, indicating that berberine and glipizide have a good synergistic effect (Li and Liu, 2007). Diarrhea and other gastrointestinal symptoms are common when metformin is used in the early stage, and it is contraindicated in patients with severe liver and kidney dysfunction. Metformin still has certain limitations as a first-line drug for the treatment of diabetes (Liao and Hao, 2020). Berberine has no hepatorenal side effects, and like metformin, it can enhance the sensitivity of insulin receptors in peripheral tissues of patients, thereby lowering blood glucose, which has a good synergistic effect (Zhu et al., 2009). Berberine can reduce the intestinal absorption of glucose and reduce postprandial hyperglycemia, as a new α-glycophorin inhibitor, berberine itself has a certain bactericidal and bacteriostatic effect, which can avoid the side effects such as abdominal pain and diarrhea caused by the fermentation of intestinal (mainly colon) flora due to the inhibition of carbohydrate absorption (Li et al., 2008). Exogenous insulin must be used by injection, which is extremely inconvenient, and excessive use will also aggravate obesity and insulin resistance, thereby increasing the incidence of complications such as coronary heart disease and hypertension. And exogenous insulin also has the potential risk of causing hypoglycemia (Lang and Zhu, 2016; Li et al., 2021). In comparison, berberine can increase insulin sensitivity, reduce insulin dosage, while helping to reduce fat and control body weight (Li et al., 2008). To sum up, berberine is a highly effective and low-risk alternative and synergistic treatment regimen, which has significant advantages and broad application prospects.

### 4.3 Glucose-lowering effects of BBR and recommendations for future studies

10.7% of emergency hospitalizations in adults 65 and older were due to OHAs. Hypoglycemia accounted for 94.6% of emergency hospitalizations for endocrine drugs (Budnitz et al., 2011). It occurs in T2DM patients, when measurable glucose concentration is less than 3.9 mmol/L (Draznin et al., 2022). In clinical practice, treatment of T2DM should minimize the risk of hypoglycemia. In OHAs, sulfonylureas and glinides can close the K_ATP_ channel, depolarize the pancreatic β-cell membrane and lead to calcium influx and insulin release (Lv et al., 2020). Among the adverse events caused by sulfonylureas, hypoglycemia is the most common one. In addition, glinides also having side effects of hypoglycemia (Klein-Schwartz et al., 2016; Garber et al., 2020; Lv et al., 2020). As an insulin secretory agent, BBR has glucose-lowering effects that is dependent on hyperglycemia, meaning that it may not lead to hypoglycemia (Zhao et al., 2021). In the two meta-analyses from 2019 to 2021 (Liang et al., 2019; Guo et al., 2021), the reviewers compared the glucose-lowering efficacy of BBR and the OHAs commonly used in clinical practice in the subgroup analysis, and there was no significant difference in the effect of the two kinds of drugs on FPG, HbA1c, and 2hPBG. This indicates that the glucose-lowering effects of BBR is similar to that of existing OHAs, and may have the advantage of not causing hypoglycemia.

Among the ion channels associated with glucose-stimulated insulin secretion (GSIS) from pancreatic islet β cells, the three most important ones are K_ATP_ channels, voltage-gated k^+^(k_v_) channels and voltage-gated Ca2^+^channels (VGCC) (Yang et al., 2014). In addition, voltage-gated sodium channels (VGSC) and transient receptor potential melastatin 2 (TRPM2) by acetylcholine also play important roles in insulin secretion (Rolland et al., 2002; Yang et al., 2014; Kakei et al., 2016). When blood glucose concentration increases, glucose is taken up into the pancreatic β cells through glucose transporters (GLUT) and converted to ATP during aerobic oxidation. If the ATP/adenosine diphosphate (ADP) ratio rises to a sufficiently high level, K_ATP_ channels will be closed, resulting in the inhibition of potassium outflow and the slow depolarization of pancreatic islet β-cell membrane. The membrane potential, which reaches the threshold of action potentials, activates VGCC to let the extracellular Ca2^+^ flow in. The increase of intracellular Ca2^+^ promotes insulin secretion. Thereafter, Kv channels will be activated to repolarize cell membrane. Then, the repolarization will close VGCC and stop Ca2^+^ influx and insulin secretion (Yang et al., 2014; Sun H. Z. et al., 2022).

There are many Kv channels expressed in human islets, such as Kv1.6, Kv2.1, Kv3.2, Kv11.1, Kv11.2 and so on (Finol-Urdaneta et al., 2012; Jacobson and Shyng, 2020). The gene, KCNH6, encodes the Kv11.2 channel, which is the target of BBR (Jacobson and Shyng, 2020; Zhao et al., 2021). BBR binds to KCNH6 potassium channel, making it close faster, and then inhibits the repolarization of pancreatic islets β-cell membrane. This will prolong the duration of cell membrane action potential and allow more Ca2^+^ to enter the cell through VGCC, so as to increase the secretion of insulin (Zhao et al., 2021). The repolarization of pancreatic islets β-cell membrane is secondary to glucose-mediated K_ATP_ closure and depolarization of pancreatic islet β-cell membrane. If glucose level is low and glucose-mediated K_ATP_ channel closure does not occur, inhibition of the KCNH6 potassium channel by BBR will not cause insulin secretion. That is to say, the effect of BBR in promoting insulin secretion is hyperglycemic dependent. So, BBR does not affect fasting insulin and blood glucose levels. In Zhao’s hyperglycemic clamp experiment involving 15 healthy men, BBR did not change subjects’ fasting blood glucose, fasting insulin, and fasting C-peptide compared with placebo (Zhao et al., 2021). As another Kv channel similar to KCNH6, Kv1.7 channel has been experimentally studied for its role in promoting insulin secretion. In a study, the researchers used Conkunitzin-S1 to selectively inhibit Kv1.7 channels in rat pancreatic islets. It was found that Conkunitzin-S1 increased insulin secretion at 10 and 16 mM glucose concentrations compared to 0 mM glucose concentrations (Finol-Urdaneta et al., 2012).

According to our meta-analysis, BBR has glucose-lowering effects, which is related to baseline FPG and HbA1c. However, how baseline glucose levels affect the therapeutic effect of BBR on diabetes and what is the specific mechanism still remains unclear. In addition, previous studies have found that glyburide, as an inhibitor of K_ATP_ channel, differently promoted insulin secretion in different blood glucose concentrations (Ligtenberg et al., 1997; Riefflin et al., 2015). As an inhibitor of Kv channel, the role of BBR in promoting insulin secretion in different blood glucose concentrations remains unclear. Subsequent studies should explore the different insulin-stimulating effects of BBR in different levels of blood glucose concentrations, such as low, moderately elevated, and high levels, and the different therapeutic effects of BBR on FPG, HbA1c, 2hPBG and other indicators reflecting diabetes in different baseline glucose levels.

When stimulated by glucose, insulin is secreted from pancreatic islet β cells in two phases. In the first phase, insulin secretion reaches a rapid peak and then declines. Then it will enter the second phase and gradually increase again (Ligtenberg et al., 1997). Early insulin secretion usually refers to the insulin secretion between 0 and 30min, including the first phase and part of the second phase. It is very important to control postprandial blood glucose (Pratley and Weyer, 2001). It is well known that sulfonylureas and glinides are the most commonly used insulin secretory agents in clinical practice. As a mealtime blood glucose regulator, the main function of glinides is to promote early insulin secretion. Sulfonylureas act slowly, so they are often not used as dietary glucose regulators. In a study (Zhao et al., 2021), BBR promoted insulin secretion in the first phase, but the result was not statistically significant. BBR also promoted insulin secretion in the second phase and overall total secretion. This result was statistically significant. In our meta-analysis, BBR reduced both FPG and 2hPBG. Therefore, BBR may be an insulin secretory agent that mainly reduces FPG but also has the effect of reducing PBG. More studies are needed to verify this hypothesis.

There was a question that our study has not been able to resolve. Firstly, Yang’s study found that, on insulin secretion, the effect of BBR depends on high blood glucose concentration. BBR had no significant effect on fasting blood glucose, fasting insulin, or fasting C-peptide levels. It is worth noting that the subjects in this trial were healthy men with normal glucose tolerance (Zhao et al., 2021). What is the effect of BBR when used in diabetic patients? In our meta-analysis, the results showed that BBR can reduce FPG in patients with T2DM. The difference between the results of our meta-analysis and those of Yang’s study may be due to the different baseline blood glucose levels of the included patients. So when will the glucose-lowering effects of BBR stop in the process of blood glucose reduction. (Di Pierro et al., 2012).

In addition to BBR, previous studies have found a variety of other herb extracts which target ion channels in the cell membrane of pancreatic islet β cells. Baicalein, a kind of flavonoid extracted from scutellaria baicalensis, can inhibit Kv channel (Guo et al., 2018). Vindoline, the alkaloids of Catharanthus roseus, can inhibit Kv2.1 channel (Yao et al., 2013). Both baicalein and vindoline can prolong the duration of cell membrane action potential and increase the secretion of insulin. Geniposide, an iridoid glycoside extracted from the fruit of Gardenia jasminoides Ellis, can inhibit Kv channel and activate VGCC, so as to make more Ca2^+^ enter the cell and increase the secretion of insulin (Zhang et al., 2016). Geniposidic acid is also one of the components of the total glycosides of Plantaginis Semen (Tong, 2019). All of these compounds–BBR, baicalein, vindoline and geniposide–are Kv channel inhibitors. When blood glucose concentration rises, K_ATP_ channels close and action potentials are generated, therefore Kv channel inhibitors increase insulin secretion by inhibiting Kv channels and increasing calcium influx. In other words, the glucose-lowering effects of Kv channel inhibitors is secondary to the increase of blood glucose concentration. Therefore, Kv channel inhibitors do not affect fasting insulin or glucose levels (Zhao et al., 2021; Sun H. Z. et al., 2022).

It is worth noting that, as the sources of BBR, baicalein and geniposide, rhizoma coptidis, scutellaria baicalensis, phellodendri chinensis and gardenia jasminoides are main herbs of Huanglian Jiedu Decoction, which have been used to treat diabetes in China for more than a thousand years (Wu et al., 2019). By further studying the mechanisms for lowering blood glucose of herbal extracts that can inhibit Kv channel, such as BBR, baicalein, vindoline, and geniposide, new insulin secretagogues may be developed that do not cause hypoglycemia.

## 5 Strengths and limitations

Compared with previous meta-analyses, our study is the first to conduct a subgroup analysis according to patients’ baseline mean FPG and HbA1c levels to investigate the source of heterogeneity. In a meta-analysis (Liang et al., 2019), through subgroup analyses, reviewers found that the intervention, patient age, daily dose of BBR, and duration of treatment led to significant heterogeneity. Our study identified that the glucose-lowering effects of BBR were also correlated with baseline mean FPG and HbA1c, which represented the patient’s blood glucose levels before the BBR intervention. Furthermore, our study resolved the problem of large heterogeneity existing in previous meta-analyses to some extent through subgroup analysis, and provided more reliable evidence for the glucose-lowering effects of BBR.

However, this review also has limitations. First, the included studies of 2hPBG had publication bias. This may affect the credibility of the meta-analysis results on 2hPBG. Secondly, the included studies were mostly conducted in China, lacking adequate global data, which weakens the extrapolation of the results. What’s more, the duration of treatment is short in some studies, which is not conducive to fully evaluating the safety of berberine in the treatment of T2DM. Additionally, the quality of the articles was uneven. Some trials did not use blinding or allocation concealment methods, which may lead to information bias due to the influence of subjective factors. But when we performed sensitivity analyses by eliminating studies with too short treatment duration (≤3w) and excluding the article of apparently poor quality from the analysis of FPG, the results of meta-analysis were not substantially changed, which suggests our findings were stable.

There are few high-quality large-scale clinical trials using BBR in the treatment of T2DM. It is suggested that relevant clinical trials in the future should follow the relevant principles of randomization, allocation concealment, and double blinding, and be described in detail in the methodology section. Large sample, multicenter, and high-quality clinical studies are needed to confirm the benefit of BBR in T2DM.

## 6 Conclusion

Our systematic review and meta-analysis showed that BBR has a glucose-lowering effect, which is related to baseline FPG and HbA1c levels of patients. The addition of BBR to lifestyle modifications or existing OHAs does not increase the incidence of total adverse events and the risk of hypoglycemia. Studies on BBR are expected to develop new glucose-lowering agents in the future.

## Data Availability

The original contributions presented in the study are included in the article/Supplementary Material, further inquiries can be directed to the corresponding authors.

## References

[B1] Bray J. J. H. Foster-Davies H. Salem A. Hoole A. L. Obaid D. R. Halcox J. P. J. (2021). Glucagon-like peptide-1 receptor agonists improve biomarkers of inflammation and oxidative stress: A systematic review and meta-analysis of randomised controlled trials. Diabetes Obes. Metab. 23 (8), 1806–1822. 10.1111/dom.14399 33830637

[B2] Budnitz D. S. Lovegrove M. C. Shehab N. Richards C. L. (2011). Emergency hospitalizations for adverse drug events in older Americans. N. Engl. J. Med. 365 (21), 2002–2012. 10.1056/NEJMsa1103053 22111719

[B3] Cao Y. (2007). Clinical Research on Multiple factor intervention of improving the insulin resistance. Master’s thesis. China: Shandong Traditional Chinese Medicine University. (In Chinese). 10.7666/d.Y1144281

[B4] Chang W. Chen L. Hatch G. M. (2015). Berberine as a therapy for type 2 diabetes and its complications: From mechanism of action to clinical studies. Biochem. Cell Biol. 93 (5), 479–486. 10.1139/bcb-2014-0107 25607236

[B5] Chang Y. C. Lin C. J. Hsiao Y. H. Chang Y. H. Liu S. J. Hsu H. Y. (2020). Therapeutic effects of bcg vaccination on type 1 diabetes mellitus: A systematic review and meta-analysis of randomized controlled trials. J. Diabetes Res. 2020, 8954125. 10.1155/2020/8954125 32309449 PMC7139880

[B6] Di Pierro F. Villanova N. Agostini F. Marzocchi R. Soverini V. Marchesini G. (2012). Pilot study on the additive effects of berberine and oral type 2 diabetes agents for patients with suboptimal glycemic control. Diabetes Metab. Syndr. Obes. 5, 213–217. 10.2147/dmso.S33718 22924000 PMC3422905

[B7] Dong H. Wang N. Zhao L. Lu F. (2012). Berberine in the treatment of type 2 diabetes mellitus: A systemic review and meta-analysis. Evid. Based. Complement. Altern. Med. 2012, 591654. 10.1155/2012/591654 PMC3478874 23118793

[B8] Dong K. L. Shang J. J. Tao L. (2017). Effect of berberine combined with metformin on serum inflammatory factors and islet function in type 2 diabetes mellitus. J. Clin. pathology 37 (07), 1418–1422. (In Chinese).

[B9] Draznin B. Aroda V. R. Bakris G. Benson G. Brown F. M. Freeman R. (2022). 6. Glycemic targets: Standards of medical care in diabetes-2022. Diabetes Care 45 (1), S83–s96. 10.2337/dc22-S006 34964868

[B10] Fan C. Li Y. Song H. P. Wang Q. (2018). Effectiveness evaluation analysis on berberine tablets combination with metformin treating type 2 diabetes. Mod. Hosp. 18 (5), 731–733. (In Chinese). 10.3969/j.issn.1671-332X.2018.05.033

[B11] Finol-Urdaneta R. K. Remedi M. S. Raasch W. Becker S. Clark R. B. Strüver N. (2012). Block of Kv1.7 potassium currents increases glucose-stimulated insulin secretion. EMBO Mol. Med. 4 (5), 424–434. 10.1002/emmm.201200218 22438204 PMC3403299

[B12] Garber A. J. Handelsman Y. Grunberger G. Einhorn D. Abrahamson M. J. Barzilay J. I. (2020). Consensus statement by the AMERICAN association of clinical endocrinologists and AMERICAN college of endocrinology on the comprehensive type 2 diabetes management algorithm - 2020 executive summary. Endocr. Pract. 26 (1), 107–139. 10.4158/cs-2019-0472 32022600

[B13] Gu Y. Zhang Y. Shi X. Li X. Hong J. Chen J. (2010). Effect of traditional Chinese medicine berberine on type 2 diabetes based on comprehensive metabonomics. Talanta 81 (3), 766–772. 10.1016/j.talanta.2010.01.015 20298851

[B14] Guo Y. Y. Liu M. M. Yang X. H. Ren L. L. Liu Z. H. Liu Y. F. (2018). Effect and mechanism of baicalein on insulin secretion in rats. Chin. Pharmacol. Bull. 34 (6), 820–824. (In Chinese). 10.3969/j.issn.1001-1978.2018.06.016

[B15] Guo J. Chen H. Zhang X. Lou W. Zhang P. Qiu Y. (2021). The effect of berberine on metabolic profiles in type 2 diabetic patients: A systematic review and meta-analysis of randomized controlled trials. Oxid. Med. Cell. Longev. 2021, 2074610. 10.1155/2021/2074610 34956436 PMC8696197

[B16] Hou K. A.-O. Wu Z. X. Chen X. Y. Wang J. Q. Zhang D. Xiao C. (2022). Microbiota in health and diseases. Signal Transduct. Target. Ther. 7, 135. (2059-3635 (Electronic)). 10.1038/s41392-022-00974-4 35461318 PMC9034083

[B17] Jacobson D. A. Shyng S.-L. (2020). Ion channels of the islets in type 2 diabetes. J. Mol. Biol. 432 (5), 1326–1346. 10.1016/j.jmb.2019.08.014 31473158 PMC7720859

[B18] Jiang W. L. Wang H. R. (2019). Effect of berberine hydrochloride tablets combined with metformin on blood glucose control and quality of life in patients with type 2 diabetes mellitus. Chronic Pathematology J. 8, 1228–1229. (In Chinese). 10.16440/j.cnki.1674-8166.20190912.004

[B19] Kakei M. Yoshida M. Dezaki K. Ito K. Yamada H. Funazaki S. (2016). Glucose and GTP-binding protein-coupled receptor cooperatively regulate transient receptor potential-channels to stimulate insulin secretion [Review]. Endocr. J. 63 (10), 867–876. 10.1507/endocrj.EJ16-0262 27321586

[B20] Klein-Schwartz W. Stassinos G. L. Isbister G. K. (2016). Treatment of sulfonylurea and insulin overdose. Br. J. Clin. Pharmacol. 81 (3), 496–504. 10.1111/bcp.12822 26551662 PMC4767194

[B21] Lan J. Zhao Y. Dong F. Yan Z. Zheng W. Fan J. (2015). Meta-analysis of the effect and safety of berberine in the treatment of type 2 diabetes mellitus, hyperlipemia and hypertension. J. Ethnopharmacol. 161, 69–81. 10.1016/j.jep.2014.09.049 25498346

[B22] Lang C. Y. Zhu K. (2016). Efficacy and safety of berberine in the treatment of type 2 diabetes mellitus. Health Guide 35, 212. (In Chinese).

[B23] Leite M. M. Dutra M. T. da Costa M. V. G. Funghetto S. S. Silva A. O. de Lima L. R. (2021). Comparative evaluation of inflammatory parameters and substitute insulin resistance indices in elderly women with and without type 2 diabetes mellitus. Exp. Gerontol. 150, 111389. 10.1016/j.exger.2021.111389 33957262

[B24] Li B. Zhu W. L. Chen K. X. (2008). Advances in the study of berberine and its derivatives. Acta Pharm. Sin. 43 (8), 773–787. (In Chinese). 18956768

[B25] Li B. Y. Min Q. Wang G. Shao K. Y. Hu W. X. (2021). Research progress of berberine in the treatment of diabetes and its complications. J. Hubei Univ. Sci. Technol. Sci. 35 (5), 448–452. (In Chinese). 10.16751/j.cnki.2095-4646.2021.05.0448

[B26] Li M. L. (2008). Clinical observation of berberine combined with metformin in the treatment of type 2 diabetes mellitus. Hubei J. Traditional Chin. Med. 30 (02), 34–35. (In Chinese).

[B27] Li Z. Y. (2014). Clinical efficacy and safety of berberine in the treatment of type 2 diabetes mellitus. Chinese-foreign women’s health (8), 226–226. (In Chinese).

[B28] Li Z. Y. (2016). The clinical effect of sulfonylurea metformin and berberine in the treatment of diabetes. China Contin. Med. Educ. 8 (26), 174–175. (In Chinese).

[B29] Li Z. Liu L. H. (2007). Effect of berberine combined with glipizide on type 2 diabetes. J. Clin. Res. 24. (In Chinese).

[B30] Liang Y. Xu X. Yin M. Zhang Y. Huang L. Chen R. (2019). Effects of berberine on blood glucose in patients with type 2 diabetes mellitus: A systematic literature review and a meta-analysis. Endocr. J. 66 (1), 51–63. 10.1507/endocrj.EJ18-0109 30393248

[B31] Liao M. Hao Y. R. (2020). Action mechanism of berberine on diabetes mellitus and its complications. Med. Recapitulate 26 (13), 2637–2642. (In Chinese).

[B32] Ligtenberg J. J. Venker C. E. Sluiter W. J. Reitsma W. D. Van Haeften T. W. (1997). Effect of glibenclamide on insulin release at moderate and high blood glucose levels in normal man. Eur. J. Clin. Invest. 27 (8), 685–689. 10.1046/j.1365-2362.1997.1710716.x 9279533

[B33] Liu H. Y. Hu Q. M. (2008). Effect of berberine on islet p-cell function in type 2 diabetes mellitus(damp-heat type). Chin. J. Inf. TCM 15 (03), 12–14. (In Chinese).

[B34] Liu L. F. (2004). Effect of berberine in adjuvant treatment of type II diabetes mellitus. J. Sichuan Traditional Chin. Med. 22 (02), 46. (In Chinese).

[B35] Liu Z. M. (2013). Effect of metformin combined with berberine in the treatment of incipient type 2 diabetes mellitus. zhejiang J. Integr. traditional Chin. West. Med. 23 (05), 380–382. (In Chinese).

[B36] Lv W. Wang X. Xu Q. Lu W. (2020). Mechanisms and characteristics of sulfonylureas and glinides. Curr. Top. Med. Chem. 20 (1), 37–56. 10.2174/1568026620666191224141617 31884929

[B37] Nagy L. Marton J. Vida A. Kis G. Bokor E. Kun S. (2018). Glycogen phosphorylase inhibition improves beta cell function. Br. J. Pharmacol. 175 (2), 301–319. 10.1111/bph.13819 28409826 PMC5758390

[B38] Pratley R. E. Weyer C. (2001). The role of impaired early insulin secretion in the pathogenesis of Type II diabetes mellitus. Diabetologia 44 (8), 929–945. 10.1007/s001250100580 11484070

[B39] Rashidi H. Namjoyan F. Mehraban Z. Zakerkish M. Ghaderian S. B. Latifi S. M. (2018). The effects of active ingredients of barberry root (berberine) on glycemic control and insulin resistance in type 2 diabetic patients. Jundishapur J. Nat. Pharm. Prod. 13 (1), 64180. 10.5812/jjnpp.64180

[B40] Riefflin A. Ayyagari U. Manley S. E. Holman R. R. Levy J. C. (2015). The effect of glibenclamide on insulin secretion at normal glucose concentrations. Diabetologia 58 (1), 43–49. 10.1007/s00125-014-3399-1 25297572

[B41] Rolland J. F. Henquin J. C. Gilon P. (2002). G protein-independent activation of an inward Na(+) current by muscarinic receptors in mouse pancreatic beta-cells. J. Biol. Chem. 277 (41), 38373–38380. 10.1074/jbc.M203888200 12161432

[B42] Sheng Z. X. (2009). The level of serum inflammatory factors in patients with T2DM and the impact of intervention after Berberine treatment. Master’s thesis. China: Sun Yat-Sen University. (In Chinese).

[B43] Sun H. Saeedi P. Karuranga S. Pinkepank M. Ogurtsova K. Duncan B. B. (2022a). IDF Diabetes Atlas: Global, regional and country-level diabetes prevalence estimates for 2021 and projections for 2045. Diabetes Res. Clin. Pract. 183, 109119. 10.1016/j.diabres.2021.109119 34879977 PMC11057359

[B44] Sun H. Z. Lei S. H. Gong Y. C. Yao L. H. (2022b). Regulation of ion channels on insulin secretion in islet β cells. Life Sci. Res. 26 (01), 59–66. (In Chinese). 10.16605/j.cnki.1007-7847.2020.11.0262

[B45] Sun S. P. (2017). Effect of Berberine on the serum levels of IL-10, IL-6 and CRP in patients with type 2 diabetes mellitus. J. Changchun Univ. Chin. Med. 33 (03), 431–433. (In Chinese).

[B46] Tokajuk A. Krzyżanowska-Grycel E. Tokajuk A. Grycel S. Sadowska A. Car H. (2015). Antidiabetic drugs and risk of cancer. Pharmacol. Rep. 67 (6), 1240–1250. 10.1016/j.pharep.2015.05.005 26481548

[B47] Tong R. C. (2019). Study on hypoglycemic and hypotensive effects and mechanisms of total glycosides of Plantaginis semen. Doctoral thesis. China: Shanghai University of Chinese Medicine. (In Chinese). 10.27320/d.cnki.gszyu.2019.000006

[B48] Wang W. (2008). Effect of berberine on blood glucose, blood lipid and serum leptin of primary type 2 diabetes mellitus patients. Master’s thesis. China: Shanxi Medical University. (In Chinese).

[B49] Wei C. Deng X. G. (2021). Effect of berberine combined with metformin and acarbose in the treatment of newly diagnosed type 2 diabetes mellitus. Contemp. Med. Symp. 19 (11), 139–140. (In Chinese). 10.3969/j.issn.2095-7629.2021.11.084

[B50] Wu Q. H. Li B. T. Tu J. (2019). [Compound traditional Chinese medicine in treatment of diabetes]. J. Chin. Materia Medica 44 (6), 1104–1109. (In Chinese). 10.19540/j.cnki.cjcmm.20181212.001 30989971

[B51] Wu M. Yang S. Wang S. Cao Y. Zhao R. Li X. (2020). Effect of berberine on atherosclerosis and gut microbiota modulation and their correlation in high-fat diet-fed ApoE-/- mice. Front. Pharmacol. 11, 223. 10.3389/fphar.2020.00223 32231564 PMC7083141

[B52] Xiang W. Huang X. J. Huang G. X. (2011). A study of two anti-inflammatory drugs in patients with incipient type 2 diabetes. J. Pract. DIABETOLOGY 07 (2), 51–52. (In Chinese).

[B53] Xing Y. Liu X. Lin Y. Zhang Y. (2017). Progress in pharmacological effects and clinical applications of berberine. Chin. J. Pharmacol. Toxicol. 31 (6), 491–502. (In Chinese). 10.3867/j.issn.1000-3002.2017.06.001

[B54] Xu F. J. (2008). Effects of berberine and piogntazone oil type 2 diabetes acute insulin secretion and adiponecttn. Hainan Med. 19 (5). (In Chinese).

[B55] Xue S. X. Xu J. Q. Tie L. L. (2012). Efficacy of sulfonylureas combined with berberine metformin in the treatment of diabetes. Shaanxi J. Tradit. Chin. Med. 33 (10), 1335–1336. (In Chinese).

[B56] Yang D. (2008). Effect on different model intervention of improving the 2 diabetes insulin resistance. Master’s thesis. China: Shandong Traditional Chinese Medicine University. (In Chinese). 10.7666/d.D459316

[B57] Yang L. (2010). Clinical observation of berberine hydrochloride combined with acarbose in the treatment of type 2 diabetes. Chin. J. Prim. Med. Pharm. 24, 3422–3423. (In Chinese).

[B58] Yang S. N. Shi Y. Yang G. Li Y. Yu J. Berggren P. O. (2014). Ionic mechanisms in pancreatic β cell signaling. Cell. Mol. Life Sci. 71 (21), 4149–4177. 10.1007/s00018-014-1680-6 25052376 PMC11113777

[B59] Yang X. Liu Z. M. Yang H. J. (2020). Clinical study of berberine hydrochloride tablets combined with metformin in the treatment of incipient type 2 diabetes mellitus. Shanghai J. Traditional Chin. Med. 54. (In Chinese).

[B60] Yao J. R. Xie D. H. (2015). Clinical observation of triple therapy in the treatment of type 2 diabetes mellitus. China Pharm. 29, 4107–4109. (In Chinese). 10.6039/j.issn.1001-0408.2015.29.26

[B61] Yao X. G. Chen F. Li P. Quan L. Chen J. Yu L. (2013). Natural product vindoline stimulates insulin secretion and efficiently ameliorates glucose homeostasis in diabetic murine models. J. Ethnopharmacol. 150 (1), 285–297. 10.1016/j.jep.2013.08.043 24012527

[B62] Ye L. N. (2021). Analysis of the value of sulfonylureas and berberine metformin in the treatment of diabetes. Living Sci. 24 (18), 263. (In Chinese). 10.3969/j.issn.1672-9714.2021.18.263

[B63] Ye W. P. (2010). Clinical efficacy of berberine treatment of diabetes. Mod. Hosp. 10 (8), 9–10. (In Chinese).

[B64] Yin S. L. (2011). Effects of berberine hydrochloride on blood glucose, insulin and blood lipid in newly diagnosed type 2 diabetes mellitus. Acta Acad. Med. Xuzhou 31 (1), 40–41. (In Chinese).

[B65] You S. Liu B. (2007). Clinical observation on the effect of berberine on urinary type IV collagen in diabetic patients. Chin. Hosp. Pharm. J. 6, 793–795. (In Chinese).

[B66] Yu J. W. (2020). Study on the effect of berberine hydrochloride combined with metformin on hypoglycemic effect and islet function in patients with T2DM. Master’s thesis. China: Hangzhou Normal University. (In Chinese).

[B67] Zhang H. (2017). Analysis of the value of sulfonylureas and berberine metformin in the treatment of diabetes. DIABETES NEW WORLD 20 (24), 82–83. (In Chinese).

[B68] Zhang X. G. Chen R. X. (2017). Efficacy of sulfonylureas combined with berberine metformin in the treatment of diabetes. Guidel. Health Care 25, 252. (In Chinese).

[B69] Zhang Y. D. Yuan X. (2012). Clinical observation of berberine in the treatment of type 2 diabetes. J. HEZE Med. Coll. 24 (02), 27–28. (In Chinese).

[B70] Zhang Y. Li X. Zou D. Liu W. Yang J. Zhu N. (2008). Treatment of type 2 diabetes and dyslipidemia with the natural plant alkaloid berberine. J. Clin. Endocrinol. Metab. 93 (7), 2559–2565. 10.1210/jc.2007-2404 18397984

[B71] Zhang Y. Ding Y. Zhong X. Guo Q. Wang H. Gao J. (2016). Geniposide acutely stimulates insulin secretion in pancreatic β-cells by regulating GLP-1 receptor/cAMP signaling and ion channels. Mol. Cell. Endocrinol. 430, 89–96. 10.1016/j.mce.2016.04.020 27126219

[B72] Zhao M. M. Lu J. Li S. Wang H. Cao X. Li Q. (2021). Berberine is an insulin secretagogue targeting the KCNH6 potassium channel. Nat. Commun. 12 (1), 5616. 10.1038/s41467-021-25952-2 34556670 PMC8460738

[B73] Zhou R. Xiang C. P. Zhang J. J. Yang H. J. (2020). Research progress on chemical compositions of Coptidis Rhizoma and pharmacological effects of berberine. China J. Chin. Materia Medica 45 (19), 4561–4573. (In Chinese). 10.19540/j.cnki.cjcmm.20200527.202 33164419

[B74] Zhu G. C. Zhang C. S. Kang S. K. (2009). Effect of berberine combined with metformin in the treatment of type II diabetes mellitus. Chin. J. Misdiagn 9 (04), 788–789. (In Chinese).

[B75] Zhu J. X. Wang X. Y. Lv W. Q. Ji X. Z. (2015). Gliclazide combined with berberine in the treatment of type 2 diabetes mellitus. zhejiang J. Integr. traditional Chin. West. Med. 25 (10), 915–917. (In Chinese).

[B76] Zhu Y. Q. Jiang H. Sha W. J. Lei T. (2020). Effect of berberine on hypoglycemic effect and gut microbiota in patients with newly diagnosed type 2 diabetes mellitus. J. TONGJI Univ. Sci. 41 (04), 467–472. (In Chinese).

